# Brazilian Nutritional Consensus in Hematopoietic Stem Cell Transplantation: Graft- *versus* -host disease

**DOI:** 10.31744/einstein_journal/2020AE4799

**Published:** 2020-03-13

**Authors:** Andréa Z Pereira, Afonso Celso Vigorito, Alessandro de Moura Almeida, Alexandre de Almeida Candolo, Ana Carolina Leão Silva, Ana Elisa de Paula Brandão-Anjos, Bianca Laselva de Sá, Catarina Lôbo Santos de Souza, Cláudio Galvão de Castro, José Salvador Rodrigues de Oliveira, Juliana Bernardo Barban, Elaine Maria Borges Mancilha, Juliana Todaro, Lilian Pinheiro Lopes, Maria Cristina Martins de Almeida Macedo, Morgani Rodrigues, Paulo Cesar Ribeiro, Roberto Luiz da Silva, Telma Sigolo Roberto, Thays de Cássia Ruiz Rodrigues, Vergilio Antonio Rensi Colturato, Eduardo José de Alencar Paton, George Maurício Navarro Barros, Rosana Ducatti Souza Almeida, Maria Claudia Rodrigues Moreira, Mary Evelyn Flowers

**Affiliations:** 1 Hospital Israelita Albert Einstein São PauloSP Brazil Hospital Israelita Albert Einstein , São Paulo , SP , Brazil .; 2 Universidade Estadual de Campinas CampinasSP Brazil Universidade Estadual de Campinas , Campinas , SP , Brazil .; 3 Universidade Federal da Bahia SalvadorBA Brazil Universidade Federal da Bahia , Salvador , BA , Brazil .; 4 Hospital de Câncer de Barretos, BarretosSP Brazil Hospital de Câncer de Barretos, Barretos , SP , Brazil .; 5 Hospital Sírio-Libanês São PauloSP Brazil Hospital Sírio-Libanês , São Paulo , SP , Brazil .; 6 Fundação Hospital Amaral Carvalho JaúSP Brazil Fundação Hospital Amaral Carvalho , Jaú , SP , Brazil .; 7 Complexo Hospitalar Universitário Professor Edgard Santos Universidade Federal da Bahia SalvadorBA Brazil Complexo Hospitalar Universitário Professor Edgard Santos , Universidade Federal da Bahia , Salvador , BA , Brazil .; 8 Santa Casa de Misericórdia de Porto Alegre Porto AlegreRS Brazil Santa Casa de Misericórdia de Porto Alegre , Porto Alegre , RS , Brazil .; 9 Universidade Federal de São Paulo São PauloSP Brazil Universidade Federal de São Paulo , São Paulo , SP , Brazil .; 10 Instituto Brasileiro de Controle do Câncer São PauloSP Brazil Instituto Brasileiro de Controle do Câncer , São Paulo , SP , Brazil .; 11 Hospital São Luiz São PauloSP Brazil Hospital São Luiz , São Paulo , SP , Brazil .; 12 Oncobio Nova LimaMG Brazil Oncobio , Nova Lima , MG , Brazil .; 13 Instituto Nacional de Câncer José Alencar Gomes da Silva Rio de JaneiroRJ Brazil Instituto Nacional de Câncer José Alencar Gomes da Silva - INCA, Rio de Janeiro , RJ , Brazil .; 14 Seattle Cancer Care Alliance SeattleWA United States Seattle Cancer Care Alliance , Seattle , WA , United States .

**Keywords:** Nutrition, Graft *versus* host disease, Hematopoietic stem cell transplantation

## Abstract

The Brazilian Consensus on Nutrition in Hematopoietic Stem Cell Transplantation: Graft- *versus* -host disease was approved by *Sociedade Brasileira de Transplante de Medula Óssea* , with the participation of 26 Brazilian hematopoietic stem cell transplantation centers. It describes the main nutritional protocols in cases of Graft- *versus* -host disease, the main complication of hematopoietic stem cell transplantation.

## EMATOPOIETIC STEM CELL TRANSPLANTATION

Over the past 20 years, research on hematopoietic stem cell transplantation (HSCT) has enabled better donor selection, reduced toxicity from conditioning, reduced intensity regimens and improved supportive care, with reduced post-transplantation complications, thus increasing the survival of transplant recipients. ^( 1 , 2 )^

Graft- *versus* -host disease (GVHD) is the major cause of allogeneic HSCT-related morbidity and mortality, accounting for a major impact on the quality of life of these patients. Approximately 30% to 50% of allogeneic transplant recipients have post-HSCT GVHD. ^( 3 )^ The global survival rate of patients with GVHD, particularly the chronic form, is 72% at 1 year, and 55% at 5 years. ^( 2 )^

## PATHOPHYSIOLOGY OF GRAFT- *VERSUS* -HOST DISEASE

Graft- *versus* -host disease is caused by activation of T cells that recognize host antigens as non-self, causing an autoimmune reaction in recipient organs, such as skin, lungs, liver, gastrointestinal tract (GIT), thymus, hematopoietic system and possibly even the central nervous system. ^( 1 , 2 )^

Severe acute GVHD (a-GVHD) is characterized by severe skin, gastrointestinal and hepatic lesions, whereas the chronic form is associated with progressive ulcerative mucosal damage, and systemic lesions to other organs, such as the skin and lungs. ^( 3 )^

Chronic GVHD (c-GVHD) has more characteristics of alloimmunity and immunodeficiency. Very similar to a-GVHD, c-GVHD is also induced by donor immune cells, but its pathophysiology is less well understood. Although T lymphocytes are considered the key factor in their development, recent data reveal that B cells also have an important role.

Classically, the development of GVHD can be divided into three phases: ^( 3 )^ the first phase consists of injury to recipient’s tissues by agents used in the aggressive conditioning regimens necessary to prevent recurrence of neoplastic diseases and graft rejection. Although other organs may be affected, with varying degrees of severity, the hematopoietic system and GIT are more susceptible to this toxicity.

The second phase in the development of GVHD consists of activation of T lymphocytes by host antigen-presenting cells, and later by donor antigen-presenting cells, that acquire effector helper T cell functions and secrete cytokines, which subsequently accelerate the immune activation. ^( 4 , 5 )^

In the third phase of GVHD pathogenesis, the immunological activation of cytotoxic effector functions of mediator cells, such as CD 81+ T cells, causes direct lesions in the characteristic GVHD target cells in organs like liver, skin and GIT. ^( 6 , 7 )^

In search of more knowledge about GVHD and how to better control it, a consensus was reached in 2005 with the formation of a working group of the National Institutes of Health (NIH). It defined that the clinical presentation, and not time, is considered the most important aspect for the diagnosis and differentiation between a-GVHD and c-GVHD. Some signs and symptoms are similar in both conditions; the differences, however, are striking and allow the definition of two distinct clinical syndromes.

## ACUTE GRAFT *VERSUS* HOST-DISEASE

A-GVHD primarily affects the skin, liver, and GIT. On skin, coalescent erythematous maculopapular lesions are observed, characteristically in the plantar region and the palm. The onset of hepatic GVHD may be heralded by increased liver enzymes and signs of cholestasis on laboratory tests. Less specific gastrointestinal symptoms are diarrhea, nausea and vomiting. This variety of symptoms is widely diverse in severity. ^( 2 - 4 )^

These conditions can be extremely aggressive, leading, for example, to laceration of the intestinal mucosa and its fecal elimination associated with secondary hemorrhages. However, there often are mild conditions that require invasive and often inconclusive differential diagnosis. ^( 2 - 4 )^ For this reason, a-GVHD was staged ( Table 1 ) to establish severity criteria ( Table 2 ) and to standardize an evaluation method in universal academic papers.

**Table 1:** Graft- *versus-* host disease organ staging categories ( ^2^ - ^4^ )

Stage	Skin findings	Liver findings	Intestinal findings
+	Maculopapular rash on <25% of body surface	Bilirubin: 2-3mg/dL	Persistent diarrhea
			(500-1,00mL) and nausea
++	Maculopapular rash on 25%-50% of body surface	Bilirubin: 3-6mg/dL	Diarrhea (1,000-1,500mL)
+++	Generalized erythroderma	Bilirubin: 6-15mg/dL	Diarrhea (>1,500mL)
++++	Peeling and blistering	Bilirubin: >15mg/dL	Pain with or without obstruction

**Table 2:** Acute Graft- *versus* -host disease global staging categories ( ^2^ - ^4^ )

Grade/stage	Skin	Liver	Intestine	Functional disorder
0 (none)	0	0	0	0
I (mild)	+ to ++	0	0	0
II (moderate)	+ to +++	+	+	+
III (severe)	++ to +++	++ to +++	++ to +++	++
IV (life-threatening)	++ to ++++	++ to ++++	++ to ++++	+++

## CHRONIC GRAFT- *VERSUS* -HOST-DISEASE

Chronic Graft- *versus* -host-disease is a clinical-pathological syndrome that involves many organs and systems, closely resembling autoimmune diseases.

Efforts have been made to identify risk factors associated with increased morbidity and mortality in patients with GVHD. Identified variables included multi-organ or local involvement, poor performance status, thrombocytopenia at diagnosis, defined as platelet count below 100,000/µL, progressive onset of c-GVHD, elevated bilirubin levels, and extensive skin involvement (involvement greater than 50% of body surface). ^( 2 , 8 , 9 )^

In 2005, the NIH developed a project to reach a consensus on the criteria that should be used in c-GVHD clinical studies. ^( 10 , 11 )^ The characteristics used in the diagnosis were standardized, as well as the methods for scoring the organs involved and for the global severity assessment. ^( 8 , 12 )^

These criteria, revised in 2014, are useful for a better analysis of the incidence of c-GVHD, and for assessing the severity of the organ or site involvement, isolated or combined, and the influence on transplant-related mortality (TRM). According to the NIH consensus, diagnostic signs and symptoms refer to manifestations that establish the presence of c-GVHD without the need for tests or the evidence of other organs affected ( Table 3 ). Distinct signs and symptoms refer to those manifestations that are not commonly found in c-GVHD, but are insufficient to establish an accurate diagnosis of c-GVHD without further testing or the involvement of other organs. Other characteristics define rare, controversial and non-specific manifestations of c-GVHD and cannot be used to confirm the diagnosis of c-GVHD. ^( 10 , 11 )^

**Table 3:** Signs and symptoms related to chronic Graft- *versus* -host-disease ( ^10^ , ^11^ )

Organ or site	Diagnostic (sufficient to establish diagnosis of c-GVHD)	Characteristic (present in GVHD but not sufficient to establish diagnosis)	Other characteristics	Common to a-GVHD and c-GVHD
Skin	Poikiloderma	Depigmentation	Depigmentation	Erythema
	Lichen planus		Excessive or absent sweating	Maculopapular rash
	Sclerotic changes		Ichthyosis	Pruritus
	Morphea		Keratosis pilaris Hypopigmentation	
	Lichen sclerosus			
			Hyperpigmentation	
			Hiperpigmentação	
Nail		Dystrophy		
		Longitudinal grooves		
		Onycholysis		
		Pterygium unguis		
		Nail drop (usually symmetrically)		
Scalp and hair		Total alopecia or alopecia areata after post-chemotherapy recovery	Thinning of hair not explainable by other causes	
		Papulosquamous lesions	Early white hair	
Mouth	Lichen-type changes	Xerostomia		Gingivitis
	Hyperkeratotic plates	Mucocele		Mucositis
	Restriction of mouth opening by sclerosis	Mucosal atrophy		Erythema
		Pseudomembranes		Pain
		Ulcers		
Eye		Dry eye and eye pain	Photophobia	
		Healing conjunctivitis	Periorbital hyperpigmentation	
		Dry keratoconjunctivitis (Schrimer test <5mm/5 minutes)	Blepharitis	
		Keratitis punctata in confluent areas		
Genitals	Lichen planus	Erosions		
	Vaginal stenosis	Fissures		
		Ulcers		
GIT	Esophageal web		Exogenous pancreatic insufficiency	Anorexia
	Stricture or stenosis in the proximal third of the esophagus			Nausea
				Vomiting
				Diarrhea
				Weight loss
Liver				Total bilirubin and alkaline phosphatase
				>twice above the normal limit
				ALT or AST > twice the upper limit
Lungs	Bronchiolitis obliterans diagnosed with biopsy	Bronchiolitis obliterans diagnosed with pulmonary function test or chest computed tomography		BOOP
Muscle, fascia, joints	Fasciitis	Myositis or polymyositis	Edema	
	Joint contractures secondary to sclerosis		Cramps	
	Joint stiffness		Arthralgia or arthritis	
Hematopoietic and immune systems				Thrombocytopenia
				Eosinophilia
				Lymphopenia
				Hypo- or hypergammaglobulinemia
				Autoantibodies (AIHA and ITP)
Other				Pleural or pericardial effusion
				Ascites
				Peripheral neuropathy
				Nephrotic syndrome
				Myasthenia gravis
				Cardiomyopathy or cardiac conduction defects

c-GVHD: chronic Graft *versus* host disease; GVHD: Graft- *versus* -host disease; a-GVHD: acute Graft- *versus* -host disease; ALT: alanine aminotransferase; AST: aspartate aminotransferase; BOOP: Bronchiolitis obliterans with organizing pneumonia; AIHA: Autoimmune hemolytic anemia; ITP: Immune thrombocytopenia purpura.

The consensus recommends the following criteria for diagnosis of c-GVHD: ^( 8 , 9 )^ distinction from a-GVHD; presence of at least one diagnostic clinical sign of c-GVHD, or presence of at least one distinct manifestation confirmed by a relevant biopsy, according to defined histopathological criteria, laboratory tests, or radiological images, on the same organ or in other organ; and exclusion of other possible diagnoses.

The revised NIH 2014 classification includes eight major organs for being those most affected by the disease: skin, mouth, eyes, GIT, liver, lungs, joints, and female genital tract. The organs most affected in mild c-GVHD are skin, mouth and liver. Lung involvement in c-GVHD adds to severity of the disease, according to the consensus. Therefore, lung damage is considered a severity criterion of great importance in this classification. ^( 8 , 9 )^

To facilitate grading and establish standardized staging criteria for the disease, the commonly affected organs were scored and graded according to severity of the injury produced by c-GVHD. Each organ or site received a score from zero to 3, with zero representing no involvement, and 3 representing severe impairment. ^( 9 )^

The global severity assessment ( Table 4 ) in this consensus is based on the number of organs or sites involved and on severity of the disease in each organ. Patients are diagnosed as having mild c-GVHD when just one or two organs (except the lungs) are affected, without any clinically significant functional damage, and with a maximum score of 1 in all organs or sites. The diagnosis of moderate c-GVHD is considered when at least one organ or site presents significant clinical impairment, but without any major damage, with a maximum score of 2 in any affected organ or site, or when two, three or more organs or sites are affected, but without any clinically significant functional impairment, with a maximum score of 1 in all affected organs or sites. A score of 1 in the lungs is also considered moderate. Severe c-GVHD indicates major damage with a score of 3 in any organ or site. A score of ≥2 in the lungs is also considered severe. ^( 9 , 10 )^ All these values are recorded in a questionnaire validated by NIH and now universally used by numerous research and care centers.

**Table 4:** Graft- *versus* -host disease global severity assessment ( ^9^ , ^10^ )

Types of chronic GVHD	Classification criteria
Mild c-GVHD	1 or 2 organs involved + score in the organs involved 1 + lung score 0
Moderate c-GVHD	3 or more organs involved + score 1 in each organ or At least 1 organ (except lung) with score 2 or Lung score 1
Severe c-GVHD	At least 1 organ with score 3 OR
	Lung score 2 or 3
1. On skin: The highest score will be used for the global severity assessment	
2. In the lungs: FEV1 is used instead of the clinical score for the global severity assessment	
3. If an abnormality of an organ is unambiguously explained by a cause not associated with GVHD, the organ score will be zero for the global severity assessment.	
4. If an organ abnormality is attributed to multifactorial causes (GVHD plus other causes), the organ score will be used for the global severity assessment, regardless of the contributing causes (the organ score will not be disregarded)	

c-GVHD: chronic Graft- *versus* -host disease; GVHD: Graft- *versus* -host disease; FEV: forced expiratory volume.

There is a growing interest in studying c-GVHD among the academic community, coupled with the recent establishment of criteria that categorize the disease based on established evidence. These are the first steps on the path for a better understanding of the pathogenesis of c-GVHD.

## INTRODUCTION ON THE IMPORTANCE OF THE NUTRITIONAL STATUS IN GRAFT- *VERSUS* -HOST DISEASE

There are no clear literature data on the impact of the pre-HSCT nutritional status as a cause of higher or lower incidence of GVHD, nor on the best way to perform its assessment. ^( 13 - 16 )^ Some studies report that high rates of malnutrition ^( 17 )^ and worsening of nutritional status are associated to more severe GVHD in the GIT, mouth, and lung. ^( 18 )^

On the other hand, despite the heterogeneity of the studies, and although no one knows exactly by what mechanism this interference occurs, both obesity and malnutrition are associated with a higher risk of GVHD. ^( 19 , 20 )^

Recovering or improving the pre-HSCT nutritional status of patients may result in a better outcome. ^( 20 )^

However, the relation between GVHD and deficient states, such as vitamin deficits, is well known. ^( 14 - 16 )^

In the immediate post-transplant period (30 to 50 days), the nutritional needs reflect the increased caloric-protein intake due to conditioning, infections, a-GVHD, fever, and other metabolic complications, affecting mainly the protein balance, energy requirements, and micronutrient metabolism. ^( 21 , 22 )^

The nutritional status in a-GVHD or c-GVHD is affected by several symptoms, which are widely discussed later, such as prolonged hospital stay and high doses of corticosteroids, which profoundly affect the body composition with increased muscle loss, fluid retention, and increased visceral fat, impairing even more the nutritional status. ^( 18 , 23 - 25 )^

In the oral, pulmonary and gastrointestinal manifestations of c-GVHD, up to 29% of patients may be malnourished due to oral mucosa pain and disease activity, among other factors. ^( 18 )^ This directly influences a reduction in patient functionality and quality of life. ^( 18 )^

Graft- *versus* -host-disease is a complex condition with significant negative effects on the nutritional status, leading to a reduction in patient quality of life and functionality. ^( 18 , 25 )^ We discuss below specific topics on nutritional status and therapy in a-GVHD and in c-GVHD.

## MICRO- AND MACRONUTRIENTS IN GRAFT- *VERSUS* -HOST DISEASE

Diet therapy management depends on how GVHD manifests in the patient. Most patients start treatment with a relatively healthy diet, but this diet quickly becomes depleted. This is due to the direct toxic effects of the treatment or secondary complications, such as infections and a-GVHD itself. ^( 15 )^

Moderate to severe GVHD and the multi-drug regimens used in its prevention and treatment result in profound and prolonged immunosuppression. Despite advances in management, GVHD remains a significant problem. Patients often have high nutritional needs and present changes in carbohydrate, fat, and protein metabolism. They also find it difficult to eat for a variety of organ-dependent reasons and generally require modified diets, oral supplements, or enteral (EN) or parenteral (PNT) nutrition to prevent malnutrition. ^( 26 )^

### Nutrition recommendations: macronutrients

#### Calories

Nutritional needs in patients undergoing HSCT increase due to intense catabolism. ^( 27 )^ It is suggested that energy requirements during the early phase of HSCT and GVHD are up to 130% to 150% of estimated basal energy expenditure, which amounts to 30 to 50kcal/kg body weight per day, and these increased energy requirements contribute to patients’ weight loss. ^( 25 , 28 , 29 )^ This chronic hypermetabolic state found in these patients is a response to inflammatory cytokines (tumor necrosis factor alpha − TNF-α; interleukins – IL−1 and 6) and changes in norepinephrine and glucagon levels. ^( 25 , 30 , 31 )^ Some studies show increased serum glucagon levels leading to up to a 10% increase in basal metabolism, mainly by stimulating gluconeogenesis. ^( 30 )^ Increased norepinephrine in these cases leads to increased hepatic glucose production, and also contributes to increased basal metabolism. ^( 30 )^

A cross-sectional study with 13 patients compared the energy requirements of healthy controls with those of patients with extensive c-GVHD of skin, mucocutaneous membranes, lung, eyes and liver, using indirect calorimetry, showing a slight increase in energy requirements (1.9kcal/kg/day or 133kcal in a 70kg person), and changes in fat and carbohydrate oxidation rates. ^( 30 )^

In addition, an animal model has shown an increase in glycolysis and fatty acid metabolism for adequate alloreactive T-cell function and GVHD induction. ^( 32 , 33 )^ It is also believed that GVHD treatment itself may have effects on patients’ energy metabolism, but reports on this topic are also scarce. ^( 22 )^

In this case, we recommend using 30 to 50kcal/kg body weight per day to calculate the caloric requirements in these patients.

#### Proteins and lipids

The World Health Organization (WHO) recommends 0.83g/kg weight as an acceptable protein intake, with the maximum protein synthesis capacity reached with an intake of 1.5g/kg/day. ^( 25 )^ Although there are no well designed studies to support such reference, it is recommended that higher protein intake levels (about 1.8 to 2.5g/kg/day) are maintained in patients who have developed GVHD. ^( 25 , 29 )^ This recommendation is based on the protein loss due to exudation of the intestinal mucosa and the effect of chronic use of corticosteroids on increased protein requirements. ^29 , 34^

Lipids can be safely administered as long-chain triglycerides (LCTs) or LCT/medium-chain triglyceride mixture, which generally contribute with 30% to 40% of non-protein energy. ^( 40 , 41 )^

#### Omega 3

Omega 3 fatty acid plays a role as an immunomodulating factor. ^( 42 )^ It has been theorized that lipids could advantageously modulate GVHD by controlling cytokine production via the prostaglandin E2 pathway. Lipid manipulation is associated with glucose intolerance control. Thus, there is an increase in monounsaturated fatty acids that would replace saturated fatty acids ( Table 5 ). ^( 42 )^

**Table 5:** Recommended nutritional supplements for hematopoietic stem cell transplant recipients with Graft- *versus* -host disease at Baylor University Medical Center ( ^29^ )

Supplements	Reason for use
Multivitamin with minerals (minimum iron during first year after HSCT)	To ensure adequate vitamin and mineral resources
	Metabolism and anabolism, especially if the patient has inadequate oral intake.
Vitamin C (500mg/twice a day)	To aid in wound healing
Zinc (22mg zinc sulphate/once a day for 2 weeks)	To aid in wound healing
	To replace lost amounts in chronic disarrhea
Folic acid (1mg/day)	To meet the high requirements for red blood cell production.
	Some medications increase the metabolism or wasting of this vitamin, and therefore it needs to be replaced.
Calcium with vitamin D (dose depends on serum level)*	Interaction with cell levels for cytokine modification, reducing the GVHD inflammatory process
Omega 3 (2 g/day)	Interaction with cell levels for cytokine modification, reducing the GVHD inflammatory process

* Serum level <10ng/mL-50,000UL/week; 10-30ng/mL-10,000UL/week. HSCT: Hematopoietic stem cell transplantation; GVHD: Graft *versus* host disease.

#### Glutamine

The use of glutamine is controversial. There appear to be some benefits of oral use in reducing mucositis and GVHD, and intravenous glutamine may reduce infections. ^( 43 )^

According to the Cochrane review, glutamine not only modulates the immune system function in the digestive tract, but it can also promote intestinal healing and reduce the severity of mucositis and GIT GVHD. ^( 44 )^ The recent guideline of the European Society for Clinical Nutrition and Metabolism (ESPEN) concludes that there is insufficient evidence to recommend glutamine supplementation to reduce treatment toxicity in patients with GIT GVHD. ^( 36 )^

Therefore, due to this inconsistency in the literature, the use of glutamine in this population is not recommended.

## Nutritional recommendations: micronutrients

### Vitamin B12

The effects of GVHD in the stomach, reducing the intrinsic factor, and in the intestine, reducing vitamin B12 absorption, and the HSCT conditioning regimen resulting in crypt cell degeneration, are associated with decreased vitamin B12 ( Table 5 ). ^( 25 )^

### Vitamin C

Studies show that vitamin C plays an important role in fighting mucositis in patients with GVHD. Patients with vitamin C deficiency who received treatment with 2,000mg/month of ascorbic acid had significant visual improvements in mucositis and were able to eat again ^( 45 )^ ( Table 5 ).

### Zinc

Chronic diarrhea and malabsorption caused by GVHD can lead to zinc deficiency, which is important in maintaining the sense of taste and the integrity of the gastrointestinal mucosa. ^( 25 )^ In addition, zinc acts on healing and taste perception, and is important in the defense against intestinal infections due to the maintenance of the integrity of the intestinal mucosa. ^( 46 )^

Several studies recommended zinc supplementation in patients with GVHD, including one by Roberts et al., ^( 29 )^ who stated zinc supplementation is relevant for the treatment of recurrent lesions. Ripamonti et al., ^( 47 )^ suggested zinc supplementation (up to 3 doses of 45mg ZnSO _4_ /day) is safe and effective for treating taste perception.

In addition, experimental studies have suggested the role of this element in the activation of regulatory T cells, which may be relevant for HSCT ^( 48 )^ ( Table 5 ).

### Vitamin D

Some studies have described the presence of vitamin D deficiency in patients after HSCT, its relation with the development of GVHD, and reduction of bone mineral density. ^( 49 )^ Despite its association with inadequate nutrition, vitamin D deficiency has not been characterized as a direct complication of GVHD, ^( 50 )^ but seems to play a role in its development. Sproat et al., in a retrospective study with a small number of patients (58 transplant patients between 2000 and 2009), reported a 89.7% prevalence of hypovitaminosis D, and most of these patients had GVHD (94.8%) and used corticosteroids (98.3%). ^( 51 )^ However, other studies also found the association of low serum vitamin D (<25ng/mL) with GVHD, and also with post-transplant cytomegalovirus (CMV) reactivation. ^( 52 , 53 )^

The reduction of GVHD-related effects can be explained by the apparent role of vitamin D in the immune system, regulating the function of dendritic cells, macrophages, and B and T lymphocytes. ^( 54 - 56 )^

Patients with a-GVHD treated with corticosteroids show a tendency for a greater decrease in vitamin D. Monitoring of vitamin D levels and, if necessary, treatment for correcting its deficiency, may be indicated at regular intervals before HSCT and during the follow-up of these patients. ^( 49 )^

Calcium and vitamin D replacement in combination with bisphosphonates, or supplementation with active metabolites, such as 1,25 (OH) _2_ D3 vitamin D or 25 (OH) _3_ vitamin D have beneficial effects on bone mass and GVHD modulation. ^( 57 , 58 )^

The study of vitamin D supplementation in HSCT is relatively recent, but already offers promising results. Serum levels should be measured in the pre-HSCT and post-HSCT periods, and the deficiency should be corrected.

### Magnesium

The main change in metabolism in GVHD patients is hypomagnesemia, caused by calcineurin inhibitors, one of the most widely used drug classes for both prophylaxis and treatment of the disease. However, there are case reports of severe hypermagnesemia following the use of high magnesium laxative medications, probably associated with dehydration and high intestinal permeability seen in GVHD. ^( 59 )^

### Iron

Iron overload is a common complication of HSCT due to increased iron absorption secondary to anemia and multiple transfusions. Iron overload may increase the risk of GVHD, especially the acute form, due to the tendency to cause direct liver toxicity. In addition, ferritin appears to be a poor-prognosis marker in patients with GVHD. ^( 25 , 60 )^ The use of non-iron multivitamins is recommended in this population. ^( 61 )^

Nutritional recommendations in patients with Graft- *versus* -host disease are presented in table 6 .

**Table 6:** Nutritional recommendations in patients with Graft- *versus* -host disease

Evaluate nutritional status by a specialist
Maintain energy requirements at 30 to 50 cal/kg, and protein requirements at 1.5-2g/kg
Monitor weight and nutrient intake in the first year after transplant; patients with active GVHD need longer monitoring
Advise and monitor nutritional support specifically for patients with GVHD of the gastrointestinal tract; initiate specialized nutritional support in patients with significant gastrointestinal tract dysfunction and anorexia, who are unable to maintain adequate body weight
Supplement with multivitamins/minerals (no iron due to the risk of hemochromatosis); other supplements, like vitamin C, zinc, folic acid, and omega 3 may be beneficial
Advise the patient on nutritional aspects regarding food safety and the risk of foodborne diseases during immunosuppression

Source: Adapted from Roberts S, Thompson J. Graft-vs-host disease: nutrition therapy in a challenging condition. Nutr Clin Pract. 2005;20(4):440-50. ^(^^29^^)^ GVHD: Graft *versus* host disease.

## MOST COMMON NUTRITIONAL COMPLICATIONS IN GRAFT- *VERSUS* -HOST DISEASE

Due to the importance of this theme, we will try to review below the main nutritional complications of GVHD, both those caused by its development and those related to its therapy. The side effects related to nutritional aspects of the main medications used to treat GVHD are shown in table 7 .

**Table 7:** Main medications and immunosuppressive therapies used to treat Graft- *versus* -host disease and their nutritional and metabolic side effects

Medication/therapy	Mechanism of action	Nutritional and metabolic effects
Corticosteroids	Anti-inflammatory response, inhibits IL-1, decreases IL-2 and suppresses lymphocyte proliferation	Sodium and water retention, hyperglycemia, hypercholesterolemia, increased appetite, weight gain, bone demineralization and muscular effects
Cyclosporine/tacrolimus	Inhibits T lymphocyte proliferation/response and alters IL-2 production	Hypertension, dyslipidemia, hyperglycemia, hypomagnesemia, hyperkalemia, nephrotoxicity, neurotoxicity, nausea, vomiting, taste changes and diarrhea
Methotrexate	Antimetabolite and immunosuppressive	Anorexia, nausea, vomiting, diarrhea, stomatitis, mucositis, hepatotoxicity and nephrotoxicity
Mycophenolate mofetil	Decreases lymphocytic activation and proliferation of B and T cells; suppresses antibody formation	Nausea, vomiting, diarrhea, constipation, gastrointestinal bleeding and peripheral edema
Sirolimus	Inhibits B and T lymphocyte proliferation	Dyslipidemia, hypertension, and peripheral edema
Thalidomide	Immunosuppressive and anti-inflammatory properties	Neuropathy and constipation
Antithymocyte globulin (ATG)	Decreases circulating lymphocytes	Abdominal pain, nausea, vomiting, diarrhea, hyperkalemia, hypertension, and peripheral edema
Etarnecept	TNF-α antagonist	Abdominal pain and vomiting
Ursodeoxycholic acid	Replaces native human bile acids; decreases HLA antigen expression in hepatocytes	Nausea, vomiting, diarrhea and abdominal pain
Daclizumab	Anti-IL-2 antibody	Vomiting, edema, hypertension and hypotension
Azathioprine	Prevents cytotoxic T and B lymphocyte proliferation by inhibiting DNA and RNA synthesis	Gastrointestinal hypersensitivity, hepatotoxicity, megaloblastic anemia and pancreatitis
Hydroxychloroquine	Interferes with antigen processing and presentation, proliferation, TNF-α production and cytotoxicity	Nausea, vomiting and diarrhea
Infliximab	Anti-TNF-α antibody	Abdominal pain, nausea, vomiting
Psoralen and PUVA	Interferes with antigen presentation and pro-inflammatory cytokine production	Nausea, hepatotoxicity
Extracorporeal photopheresis	Induces alloreactive T cell apoptosis, photoinactivation of antigen presenting cells	Hypocalcemia (citrate use) and gastrointestinal disorders
Cyclophosphamide	Immunosuppressive activity and blockade of cell growth by DNA metabolite binding	Anorexia, nausea, vomiting and mucositis
Rituximab	Anti-CD20 antibody	Abdominal pain, diarrhea, nausea, vomiting, hypertension and hyperglycemia
Pentostatin	DNA synthesis block	Nausea, vomiting, fatigue, diarrhea, anorexia and stomatitis
Imatinib	PDGF-r inhibition	Nausea, fatigue, diarrhea, abdominal pain, vomiting, weight gain, hepatotoxicity, hyperglycemia and myopathy

Source: Adapted from Roberts S, Thompson J. Clinical Observations Graft-vs-Host Disease: Nutrition Therapy in a Challenging Condition. Nutr Clin Pract. 2005;20:440-50. ^(^^29^^)^ IL: interleukin; TNF-α: tumor necrosis factor alpha; HLA: human leukocyte antigen; PUVA: psolaren + ultraviolet irradiation A; PDGF-r: platelet-derived growth factor receptor.

## MECHANICAL CHANGES IN THE GASTRINTESTINAL TRACT

The GIT is involved in most patients with GVHD, and any part of the digestive tract can be affected. Although rarer, mechanical and structural changes in the digestive tract deserve to be reported due to their severity and the need for early management. ^( 62 )^

Esophageal complications are rare and include ulceration, esophageal varices and vesicular lesions. Dysphagia with severe stenosis requires esophageal dilation. ^( 62 )^

One of the most serious bowel complications is intestinal perforation; however, the most common is diarrhea. ^( 63 )^

## HANGE IN NUTRIENT ABSORPTION

The change in absorption seen in patients with GVHD may be associated with hepatic and pancreatic changes. Hepatic changes may be due to impaired excretion of bile salts and play an important role in lipid metabolism. ^( 64 )^

Pancreatic changes have already been reported in autopsies of experimental models, and are associated with involvement of GVHD; however, these changes, which may include atrophy, can also be due to some medications, such as azathioprine, cyclosporine, and corticosteroids. ^( 25 )^ The main symptoms of pancreatic exocrine insufficiency are steatorrhea, fatigue, abdominal pain, weight loss, and flatulence. Such symptoms are more frequent after transplant in patients with signs of GVHD, being more frequent among more advanced degrees of the disease. ^( 65 , 66 )^

In addition to pancreatic function, GVHD in the small bowel has also been studied as a possible cause of digestive disorders in post-transplant patients. In addition to endoscopic capsule studies, a marker being tested is citrulline. The small bowel is the main source of this amino acid in our body. Previous studies in patients without GVHD showed a correlation between reduced plasma levels of citrulline and intestinal damage. ^( 67 , 68 )^ Such findings were also described among patients with intestinal GVHD. ^( 69 )^ This amino acid has also shown to be promising in predicting GVHD, ^( 70 )^ although more literature data are needed for its use in clinical practice.

## DIARRHEA AND PROTEIN-LOSING ENTEROPATHY

Diarrhea is one of the main symptoms of low digestive tract GVHD. However, its etiology in this entity is multifactorial and may include villous atrophy, mucosal ulceration, secretory dysfunction, osmotic factors, pancreatic insufficiency, and altered intestinal transit. It is often greenish, liquid, mucous and can be voluminous. ^( 25 , 71 )^

Graft- *versus* -host-disease damage to the gastrointestinal tissue can lead to a number of problems, including dehydration, electrolyte loss, and protein-losing enteropathy. This situation is defined by an increase in alpha 1-antitrypsin (>2.2mg/g dry fecal weight) in fecal samples and occurs especially in patients with digestive tract GVHD. ^( 25 , 35 )^

Papadopoulou et al., studied a sample of 47 patients undergoing HSCT, 42 of them allogeneic. They found that 91% of diarrhea episodes were associated with protein-losing enteropathy, and the amount of protein lost was more severe among patients with GVHD (19.4mg/g) than among individuals with other causes of diarrhea, such as rotavirus, CMV infection or uncertain causes (6.7mg/g). ^( 35 )^

The amount of protein loss also appears to be correlated with severity of GVHD, especially among patients undergoing myeloablative conditioning. ^( 72 )^ In addition, GVHD patients tend to persistently increase the amount of protein lost in their stools, unlike what happens with individuals with other diarrheal disorders. ^( 34 )^

## EFFECTS ON APPETITE

In addition to the effects of the conditioning regimen and the immunosuppressive and supportive medications used, the development of GVHD may, *per se* , have an effect on appetite. Malone et al., demonstrated higher oral ingestion among patients without GVHD or grade 1 GVHD compared with others. ^( 73 )^ Graft- *versus* -host disease-associated symptoms, especially of the digestive tract, are reported as causal agents of inadequate nutrition. However, this is not so easily explained. It seems that GVHD activity itself may play a role in appetite suppression. ^( 18 )^

## HANGES IN CARBOHYDRATE AND LIPID METABOLISM

Glycemic control is important during the post-transplant period. Hyperglycemia not only impacts on immune function, but also causes damage to other tissues, such as endothelial dysfunction, elevation of proinflammatory cytokines, muscular and adipose catabolism. Theoretically, hyperglycemia may increase the level of cytokines and the risk of infectious diseases, which may lead to an increased risk of GVHD. On the other hand, GVHD may also, through inflammatory mechanisms, lead to a state of hyperglycemia. ^( 74 )^ In addition, corticosteroids used in the treatment of GVHD have hyperglycemia as one of the most common side effects. ^( 29 )^

Regarding dyslipidemia, several medications used to treat GVHD are related to the development of this complication ( Table 7 ). However, not only immunosuppressive medications affect lipid homeostasis. Liver GVHD can lead to elevations of cholesterol and triglycerides, due to the inability of bile salts and cholesterol to be excreted in the bile duct. ^( 64 )^ In addition, nephrotic syndrome, which can be a severe complication of GVHD, can also lead to significant dyslipidemia. ^( 64 )^

## LOSS OF LEAN BODY MASS AND MYOPATHY

Loss of lean body mass is frequent among patients with GVHD and a consequence of nutritional changes caused by it. Corticosteroid therapy significantly influences this complication. The development of c-GVHD seems to be an independent risk factor for the loss of lean body mass, and the likelihood is higher among those with extensive GVHD and those who required corticosteroids. ^( 75 , 76 )^

## NUTRITIONAL INTERVENTION IN ACUTE AND CHRONIC GRAFT- *VERSUS* -HOST-DISEASE

Graft- *versus* -host-disease patients have difficulty ingesting food for various reasons, depending on the organ involved. They often require dietary modifications, oral supplements, and nutritional support therapy (NST) to prevent or treat malnutrition. ^( 77 )^

According to Bassim et al., ^( 18 )^ the main indications for the onset of NST are uncontrolled nausea and vomiting, voluminous diarrhea, oral and esophageal mucosa pain, dysphagia, dysgeusia, xerostomia, anorexia, early satiety and weight loss. In particular, GIT a-GVHD and oral, gastrointestinal, and pulmonary c-GVHD produce severe malnutrition and lead to impaired patient’s functional capacity and quality of life, hence the need for early onset of NST.

Nutritional therapy is of utmost importance as a treatment support to counteract the deleterious effects of GVHD and to circumvent the adverse effects of medications. ^( 15 , 25 , 77 , 78 )^

## SYMPTOM MANAGEMENT BY NUTRITIONAL CHANGES

According to the consensus on nutrition in cancer patients of the *Instituto Nacional do Câncer José de Alenca* r, ^( 21 )^ some nutritional interventions may be directed towards improving and controlling gastrointestinal symptoms.

### Early satiety

Make the patient aware of the importance of food; perform diet fractionation (from six to eight meals/day); modify dietary fiber by cooking and/or grinding to reduce satiety (unpeeled fruit, cooked vegetables, soups and liquid juices); increase the caloric and protein density of meals; do not drink fluids during meals; use lean, cooked, shredded or minced meat in small portions; avoid high-fat foods and preparations; and prefer non-carbonated drinks. ^( 21 )^

### Diarrhea

Diet fractionation is important as well as reducing the volume of food per meal; evaluate the restriction of lactose, sucrose, gluten, fat, insoluble fiber, caffeine and theine; increase water and isotonic fluid intake to at least 3L/day; avoid flatulence-producing and hyperosmolar foods; and avoid extreme temperatures. ^( 21 )^

### Dysphagia

Accompaniment with the speech therapist, for proper modification of the diet; advise the patient on the care of dry and hard foods, and prefer soft, easily chewed and swallowed foods; drink small volumes of fluid with meals to facilitate chewing and swallowing; and keep the headboard high while eating. ^( 21 )^

### Xerostomia

Consuming at least 2L/day of water and other liquids up to 3L/day is required; stimulate the intake of more enjoyable foods; adjust food consistency according to patient acceptance; avoid consuming coffee, tea and caffeine-containing soft drinks; maintain oral hygiene and lip hydration; use lemon drops on salads and drinks; if necessary, drink fluids with meals to facilitate chewing and swallowing; suck on sugarless citrus and mint candies; season foods with herbs, avoiding excess salt and condiments; chew and suck on ice cubes made of water, coconut water and fruit juice or popsicles. ^( 21 )^

### Nausea and vomiting

It is necessary to advise a fractional diet in small volumes; give preference to drier, citric, salty and cold or frozen foods; maintain oral hygiene; avoid fasting for long periods; suck on ice cubes 40 minutes before meals; avoid fried foods and fatty foods; avoid overly sweet or strong smelling foods and preparations; have meals in airy places; do not drink liquids during meals, using them in small quantities at intervals, preferably cold ( *e.g* . popsicle); do not lie down after meals; and use ginger for its antiemetic effect, in a brew, as a spice, or added to juices. ^( 21 )^

### Anorexia

The patient should be advised about the importance of adequate food intake; fractional diet in small portions; meals with higher caloric and protein density; consume foods better tolerated and of appropriate consistency, according to patient preferences. ^( 21 )^

### Odynophagia

Meal consistency should be modified according to tolerance; improve the caloric and protein density of meals; good oral hygiene; do not consume dry, hard, citric, salty, peppery and spicy foods; avoid extreme temperatures ^( 21 )^

### Oral diet

In mild oral cavity involvement, the consumption of acidic foods should be avoided; in more severe cases with esophageal stenosis, the consistency and temperature of the meals should be modified, with preference to liquid or liquefied foods, served at a moderate or room temperature. ^( 21 , 74 )^

During treatment with high doses of glucocorticoids and/or calcineurin inhibitors, proper patient orientation is important. Frequent and small-portion meals, soluble and insoluble fiber-rich diet, high-protein diet with reduced simple and high-glycemic-index carbohydrates, sodium reduction, good water intake and adequate intake of food sources of vitamin D, calcium, magnesium and zinc are recommended, and, if necessary, a supplementation of these elements. ^( 79 , 80 )^

### Oral supplements

Regardless of the type and severity of GVHD, when the patient has a dietary intake below 70% of the energy requirements in the last 3 days, and symptoms that impair adequate nutrition, it is important to intervene with the use of high-calorie and high-protein nutritional supplements (adapted according to the phase of the restricted diet in the case of intestinal GVHD). Discontinuing oral nutritional supplementation is indicated only in the presence of hemodynamic instability, esophagitis, or severe mucositis that prevent adequate oral intake, GIT obstruction, incoercible vomiting, risk of bronchoaspiration, patient refusal, and supplemental intolerance. ^( 21 )^

## ENTERAL NUTRITION

If the food intake is below 60% of energy requirements in the last 3 days or oral use is contraindicated, EN may be prescribed. ^( 21 )^ The enteral route, if tolerable and clinically possible, may be chosen for maintaining digestive function and integrity of the mucosal barrier, preventing bacterial translocation in the digestive tract. ^( 25 )^

According to the American Society for Parental and Enteral Nutrition, ^( 77 )^ when neutrophil and platelet counts are normal and GIT is healed, NE is safe for the transition from parenteral nutritional therapy to oral diet, or when NST is necessary, in case of GVHD, among other late complications of HSCT.

According to a systematic review by Baumgartner et al., ^( 15 )^ several studies compared EN with PNT, showing superior results for the enteral route and moderate to high tolerance to the tube, and PNT is recommended only in cases of gastrointestinal insufficiency. EN is contraindicated when there are hemodynamic instability and/or worsening of abdominal pain, abdominal distension, mucositis, diarrhea, incoercible vomiting, paralytic ileus, and intestinal bleeding. ^( 21 )^

There is strong evidence indicating that early introduction of EN may decrease both the incidence and severity of GIT GVHD and may be a form of prophylaxis. In addition, EN is associated with lower infection-related mortality and shorter times of neutrophil engraftment. ^( 81 )^

## PARENTERAL DIET

Parenteral Nutrition Therapy can also be indicated for patients who have an oral diet acceptance of less than 60% to 70% of nutritional requirements for 3 consecutive days, ^( 82 )^ or in patients with energy-protein deficiency with exclusive use of EN. ^( 83 )^

American Society for Parental and Enteral Nutrition guidelines recommend oral or enteral diet, as long as possible, but in case of vomiting, incoercible diarrhea, severe mucositis, or significant malabsorption, PNT should be the preferred route. ^( 36 )^

Studies show that patients with grade III-IV GVHD receive more PNT than patients with grade I-II GVHD, and are not exempt from clinical complications related to the number of days receiving PNT. ^( 84 )^

Some precautions should be taken when prescribing and monitoring PNT. Malnourished patients at risk of feedback syndrome should receive progressive energy intake in the initial phase (first to third day), with 20% of basal energy needs. Protein may be supplied from the outset, respecting renal and liver functions. Glycemic control should be performed, maintaining blood glucose levels lower than 180mg/dL, and avoiding hypertriglyceridemia, with serum triglyceride levels below 400mg/dL. ^( 83 )^ In addition to monitoring liver function, with the measurement of AST, ALT, gamma glutamil transferase, alkaline phosphatase and bilirubin levels twice a week, the measurement of urea, creatinine, serum electrolyte (potassium, magnesium, phosphorus, calcium and sodium), total cholesterol and fractions levels should be included in routine tests. Weaning of PNT should be gradual, respecting the offer and the patient’s acceptance of oral or enteral diet. ^( 83 )^

## NUTRITIONAL MANAGEMENT IN INTESTINAL GRAFT- *VERSUS* -HOST-DISEASE

The nutritional assessment of patients with this complication can be very difficult, since many of them have fluid retention related to low serum albumin levels, which masks body weight loss. In addition, the standard treatment of GIT GVHD is corticoid therapy, which has direct effects on body composition, leading to increased body fat, decreased lean mass, water and sodium retention, hypertriglyceridemia, hypercholesterolemia, sarcopenia and bone demineralization, and this may mask the nutritional status of patients. ^( 25 )^

The goals of nutritional therapy in GIT GVHD are to provide adequate and individualized nutritional support to maintain or restore the patient’s nutritional status, control symptoms, reestablish intestinal mucosa integrity, satisfy the patient, and promote quality of life, whenever possible. ^( 25 , 85 )^

## NUTRITIONAL THERAPY IN INTESTINAL GRAFT- *VERSUS* -HOST DISEASE

### Oral diet

At the National Cancer Center Japan, a study was carried out with stepped nutritional therapy with its own protocol, and it was observed that the nutritional status of patients tended to improve with this type of therapy. However, no improvement was observed in the overall severity of GIT GVHD. ^( 86 )^

At the Seattle Cancer Care Alliance, as per the physicians´ guide, nutritional therapy is also based on this type of stepped nutritional therapy, and the clinical course of the diet occurs according to the patient’s tolerance and the symptoms presented. ^( 87 )^

The use of home-made or industrialized oral supplements can take place from step 2, when food intake does not meet the recommended nutritional needs, and should follow the same characteristics of the corresponding step of the diet and the patient’s wishes.

Based on this literature, table 8 shows how the patient’s nutritional therapy should be altered, according to symptoms, clinical course and tolerance. Whenever the patient does not tolerate a change in diet, the patient should go back to the previous step.

**Table 8:** Stepped progression of the nutritional therapy in patients with intestinal Graft- *versus-* host disease

Step	Symptoms	Nutrition therapy
1. Bowel rest	Large volume of watery diarrhea (over 1,000mL/day); intestinal cramps; serum albumin depletion; decreased intestinal transit; bowel obstruction; nausea and vomiting	PNT only
2. Introduction of oral/enteral feeding	Diarrhea volume less than 500mL/day; decreased intestinal cramps; improvement of intestinal transit time; decrease in nausea and vomiting	PNT + oral/enteral diet with characteristics: isosmotic liquid, no residues, no lactose, no acids, and low in fat
3. Introduction of solid foods	Absence or reduction of cramps and more consistent stools	Oral/enteral diet with characteristics: solid foods, without residues, without lactose, low in fat, and no gastric acids and irritants
4. Expansion of diet	Absence or reduction of cramps and more consistent stools	Oral/enteral diet (if necessary, according to the individuality of the patient) with characteristics: low in fiber, lactose, acids, gastric irritants, and fat according to the tolerance of the patient
5. Introduction of the patient’s usual diet	Absence of colic and stools of normal consistency	Oral diet with characteristics: gradual introduction and according to the patient’s tolerance of acidic foods, gastric irritants, fiber, lactose and fat

Source: Adapted from Fred Hutchinson Cancer Research Center. Long-term follow-up after hematopoietic stem cell transplant. Fred Hutchinson Cancer Research Center/ Seattle Cancer Care Alliance [Internet]. Seattle, WA; 2014 [cited 2019 June 25]. Available from: https://www.fredhutch.org/content/dam/public/Treatment-Suport/Long-Term-Follow-Up/physician.pdf; ^(^^80^^)^ Gauvreau JM, Lenssen P, Cheney CL, Aker SN, Hutchinson ML, Barale KV. Nutritional management of patients with intestinal graft- *versus* -host disease. J Am Diet Assoc. 1981;79(6):673-7. ^(^^88^^)^ PNT: parenteral nutrition therapy.

### Parenteral diet

Patients with GIT GVHD in the acute and early phase of the disease usually present diarrhea, with stool volume >1,000mL/day, making oral or enteral nutrition insufficient to meet their minimum nutritional needs. This can last for days or weeks. Thus, the most appropriate nutritional therapy would be GIT rest with fasting and the use of parenteral nutritional therapy. ^( 28 )^

The most traditional approach in the nutritional management of GIT GVHD is the administration of PNT and the initiation of oral ingestion only after the recovery of GIT symptoms. However, due to prolonged use of PNT, damage to the intestinal mucosa occurs, inducing atrophy and further intestinal dysfunction.

### Enteral diet

The introduction of oral or EN diet from step 2 should occur after diarrhea volume reduction to less than 500mL/day; decreased intestinal cramps; improvement of intestinal transit time; decreased nausea and vomiting. This gradual introduction should be prioritized because it assists in the maintenance of intestinal tropism, helps preserve mucosal barrier, and local and systemic immunity, and also prevents bacterial translocation. ^( 13 )^

The choice of oral, enteral or concomitant nutrition during the progressive stages of nutritional therapy is based on the symptoms and the possibility of oral feeding and in situations when oral diet is inadequate to meet nutritional needs.

Enteral, if chosen as a route of nutrition or supplementation, must follow the characteristics of each step. From step 2 on, the patient does not tolerate large volumes of oral and/or enteral diet, therefore PNT does not need to be suspended, in order to meet all the patient’s nutritional needs.

Some studies showed the use of EN in GIT GVHD, as compared to PNT, reduced infectious complications by preserving intestinal tropism, improving intestinal barrier function and thus decreasing the risk of bacterial translocation. ^( 89 , 90 )^ However, historically, transplant centers prefer PNT to EN, making it difficult to use it early or during HSCT.

## THE IMPORTANCE OF THE MICROBIOTA IN HEMATOPOETIC STEM CELL TRANSPLANTATION

### Intestinal microbiota

Human GIT can be populated by up to 100 trillion bacteria (for comparison, the number of cells in the human body is estimated at 10 trillion), as well as by viruses and fungi also present in considerable number and diversity, and which may be from approximately 1,000 different species in a single individual. More than 15,000 different species have already been identified in human GIT-derived samples. ^( 91 )^

Gastrointestinal tract immune system is the first line of defense against microorganisms and other ingested substances, and has evolved not only to protect against potential pathogens, but also to tolerate commensal bacteria that play a beneficial role in homeostasis, allowing symbiosis with the intestinal microbiota. The gastrointestinal immune system maintains the mucosal barrier through secretion of antimicrobial peptides and antibodies, and the commensal microbiota participates in the intestinal physiology of the host. ^( 92 , 93 )^

Intestinal exposure to bacteria is related to the recruitment of regulatory T lymphocytes (Tregs). ^( 94 , 95 )^ Tregs cells are critical for the development of an appropriate immune response to antigens within the GIT, but also influence systemic immunity. ^( 96 , 97 )^

Intestinal bacteria are responsible for the breakdown of dietary fiber and are also important for the production of a number of metabolites that function in the intestinal physiology. The best known of these metabolites are short chain fatty acids (SCFAs) such as butyrate, propionate and acetate, which serve as energy sources for intestinal epithelial cells and induce protective regulatory immune responses both locally in GIT and systemically. ^( 98 , 99 )^

### Dysbiosis

Chemotherapy and conditioning regimens alter the composition of the intestinal microbiota, causing the reduction of *Clostridium* cluster XIV and bifidobacteria strains, and the increase of *Enterococcus* . This change in the microbiota is called dysbiosis. ^( 100 - 102 )^

A specific study with patients undergoing HSCT found increased levels of proteobacteria, including *Escherichia* species, and reduced levels of *Firmicutes* , including *Blautia* species, following chemotherapy. ^( 102 )^

Nonetheless, the causal relation between chemotherapy and microbiota is difficult to establish because many of the patients studied received prophylactic antibiotics concurrently with chemotherapy.

### Intestinal microbiota and Graft- *versus* -host disease

The normal intestinal microbiota have great diversity and are dominated by anaerobic bacteria. ^( 103 )^ During hospitalization, many patients undergoing HSCT lose this diversity, and the changes that occur are influenced by both antimicrobial treatments and the development of GVHD. ^( 104 - 106 )^

The impact of microbiota on GVHD was first proposed in the 1970s, after demonstrating that mice kept under germ-free conditions developed less GIT GVHD. ^( 107 , 108 )^ Subsequent clinical studies showing promising results in intestinal decontamination of transplant patients ^( 109 , 110 )^ have not been confirmed in further research. ^( 111 , 112 )^

A large prospective study focusing on anaerobic bacterial decontamination showed a reduction in GVHD development, indicating that selective decontamination could have beneficial effects. ^( 113 )^

The loss of intestinal diversity observed in patients undergoing HSCT is generally associated with the loss of *Clostridium* species, which are known to produce short chain fatty acids from dietary fibers. ^( 114 )^

Butyrate is the preferred energy source of intestinal epithelial cells, and one study suggests that reduced amounts of butyrate are found in the intestinal epithelial cells of mice submitted to HSCT, and the addition of these fatty acids reduces intestinal lesions and the development of GVHD. ^( 115 )^

These findings are reproduced by the administration of varied species of butyrate-producing bacteria belonging to the *Clostridia* class, and a clinical study has shown that intestinal abundance of *Blautia* genus, of the *Clostridia* class, correlates with reduced mortality risk due to GVHD. ^( 116 )^

The administration of antibiotics to treat febrile neutropenia is probably the main factor affecting the changes in microbiota observed in the evolution of transplanted patients, and the choice of antibiotic regimen influences the incidence of GVHD. Imipenem-cilastatin and piperacillin-tazobactam administration was associated with higher GVHD-related mortality at 5-year follow-up, in a retrospective study. ^( 117 )^ This same study did not demonstrate the association between metronidazole and the previously reported GVHD reduction, ^( 113 )^ which may be due to a number of factors, including the use of different antibiotic combinations between studies, as well as cultural and geographical differences, which may influence intestinal flora.

The intestinal microbiota can not only predispose GVHD, but also act to recover and even prevent the disease. Intestinal damage caused by conditioning regimens causes increased intestinal permeability that allows bacteria to translocate through the enteric barrier. As a consequence, immunological stimulation by a series of pathogens and associated molecules, such as bacterial lipopolysaccharides and peptidoglycan, reinforces the cytokine-mediated inflammatory response, providing the ideal scenario for allogeneic T lymphocyte activation.

The degree of loss of intestinal microbiota diversity is a risk factor for transplant-related mortality (TRM), including GVHD mortality, infections and organ failure after HSCT. ^( 118 )^

## NUTRITION AND MICROBIOTA

The use of PNT reduces the amount of nutrients reaching the intestinal epithelium, and thus some of the changes in microbiota observed during HSCT may be due to insufficient nutrients in GIT to maintain a balanced flora. ^( 116 )^

The study that showed an association between *Blautia* reduction and GVHD also demonstrated a correlation between this finding and prolonged PNT. ^( 116 )^ These findings suggest that EN, unlike PNT, may have a beneficial effect on post-HSCT intestinal flora and perhaps accelerate patient recovery.

### Use of probiotics and prebiotics

Increasing attention has been paid to the potential of probiotics and prebiotics in the prevention and treatment of intestinal dysbiosis. Probiotics are nutritional supplements that contain a definite amount of viable microorganisms, the administration of which may confer benefits to the patient, ^( 119 )^ whereas prebiotics consist of indigestible food ingredients ( *e.g* ., indigestible fibers), which favor the growth of beneficial bacteria. ^( 119 )^

Until recently, the use of probiotics in immunosuppressed individuals was totally banned, because it was believed that as they are living bacteria, they could cause severe infectious diseases. However, this concept has been gradually modified by several studies that demonstrated, initially, their safety in this profile of patients, in addition to potential better prognostic effects.

In general, several studies showed the use of probiotics in various clinical conditions - such as inflammatory bowel diseases - is safe because they are immunosuppressed individuals, and also because it is related to the reduction of the systemic and local inflammatory response through an adequate immune response. Therefore, the indication for the use of probiotics in patients undergoing allogeneic HSCT is based on this condition. ^( 120 , 121 )^

It is known that these microorganisms may inhibit the action of external pathogens; and improve the intestinal barrier function by increasing the production of mucus and peptides with bactericidal properties, improving the structure of cell junctions between enterocytes and preventing early cellular apoptosis. ^( 120 , 121 )^

One of the strains that has its most proven safety is *Lactobacillus plantarum* (LPB). In addition to safety, it is also proven *in vitro* that its pre-HSCT use decreases GVHD severity and mortality. ^( 104 )^

According to Coehn et al., a retrospective analysis of medical records of 3,796 patients undergoing HSCT from 2002 to 2011, with the aim of identifying bloodstream infection by probiotic agents ( *Lactobacillus, Bifidobacterium, Streptococcus thermophilus* and *Saccharomyces* ), showed that only 0.5% (n=19) developed this condition one year after transplantation, and of the 19 patients, 74% received allogeneic HSCT, with 98% of bloodstream infection by *Lactobaccilus* . ^( 121 )^

In 2004, Gerbitz et al., demonstrated in an experimental study in rats that the *Lactobacillus rhamnosus* -treated group had lower mortality than the control group, especially in the recent post-HSCT period (7 to 14 days after cell infusion), and had milder GVHD manifestations. ^( 122 )^

In 2015, Laval et al., published another *in vitro* study, considering both the hypothesis that intestinal cell permeability is increased in various inflammatory bowel diseases, even GVHD, and the proven theory that certain probiotic strains can increase intestinal integrity. In this study, they demonstrated that the use of *Lactobacillus rhamnosus* can partially restore the enterocyte barrier function and also increase the production of intestinal mucosa protective dipeptides. ^( 123 )^

In 2017, Gorshein et al., demonstrated in a study of 31 allogeneic HSCT patients who received *Lactbacillus rhamnosus* at a daily dose of 10 billion strains, that their use is safe and unrelated to severe infectious complications; however, no statistical difference was found in morbidity and mortality in both groups. ^( 124 )^

According to Ladas et al., the use of LPB is subjected to rigorous microbiological analysis and therefore proven to be decontaminated at the dose of 1×108 colonies offered from day -7 to day +14. In a study involving 31 children and adolescents (2 to 17 years) undergoing allogeneic HSCT with myeloablative conditioning regimen, it was safe, so that no episode of LPB bacteremia was observed, as well as no other severe complications related to the use of LPB. ^( 125 )^

Ladas et al., also reported 70% of patients did not develop a-GVHD on day +100 and none of the patients who died on day +100 developed a-GVHD. Of the 30% who developed a-GVHD, no patient had maximum severity (grade 4). ^( 125 )^

Although the use of these treatments seems promising, further clinical studies are needed to establish the safety and efficacy of these therapies. An important aspect of the efficacy of probiotic treatment lies in the ability of ingested microorganisms to survive the acidic environment of the stomach and small intestine. Many strains of lactobacilli, including those most commonly found in common dairy products, are sensitive to low gastrointestinal pH and could not be reisolated in fecal samples after administration to humans, ^( 126 )^ making it difficult to interpret their efficacy.

The use of probiotics and prebiotics in HSCT is not yet routinely recommended.

## FECAL MICROBIOTA TRANSPLANTION

Fecal microbiota transplantation (FMT) can be used to restore damaged intestinal flora. A small series of patients with refractory or corticosteroid-dependent GVHD showed promising results; ^( 127 )^ however, larger and better controlled studies are required to determine the efficacy of FMT in the treatment of GVHD.

Fecal microbiota transplantation for the treatment of resistant *Clostridium difficile* infections is already a well-described technique in many populations. ^( 128 )^ Its use is still modest in the context of post-transplantation patients, and one of the pioneering experiments was carried out in Brazil, without major complications. ^( 129 )^ Since then, other cases have been successfully reported using familiar donors or not, and using some methods, such as retrograde enteroscopy or ingestion of capsules that open only in the jejunum, releasing the new microbiota. ^( 130 )^

At the time of publication of this consensus, FMT for immunomodulation and GVHD treatment is promising, but should only be done within well-designed clinical studies. It is necessary to understand which components of the microbiota are desirable, as well as to know the best time to perform this type of intervention. However, its use in the treatment of *Clostridium difficile* infections, although lacking randomized trials and large case series in this group of patients, can be considered in special situations, since no complications have been reported so far.

## A PRACTICAL FLOWCHART

**Figure 1:**
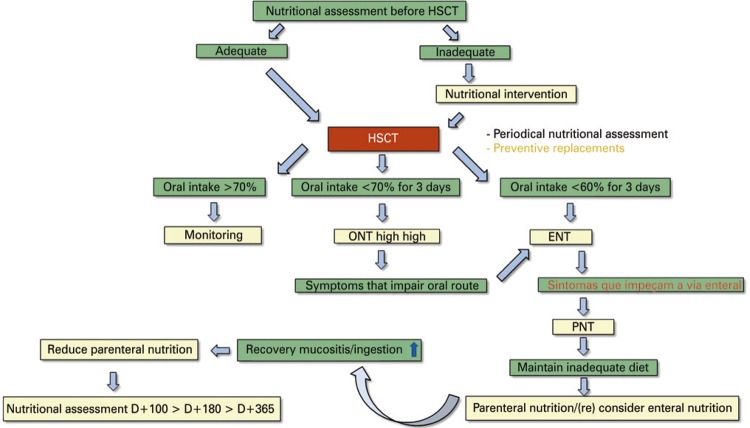
Nutritional planning for hematopoietic stem cell transplantation HSCT: hematopoietic stem cell transplantation; ONT: oral nutrition therapy; ENT: enteral nutrition therapy; PNT: parenteral nutrition therapy.

**Figure 2:**
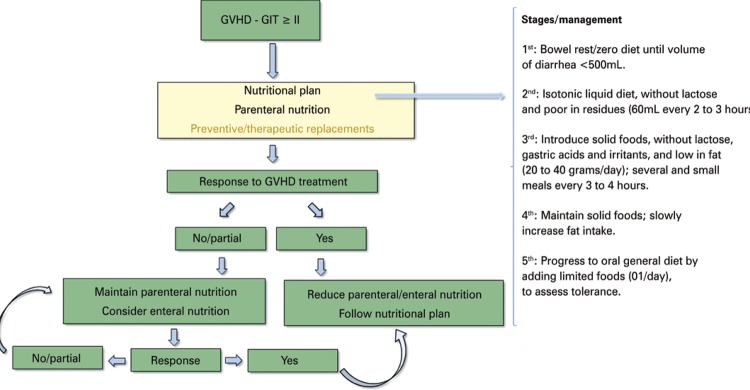
Nutritional planning for Graft- *versus* -host disease of the gastrointestinal tract GVHD: Graft- *versus* -host disease; GIT: gastrointestinal tract.

Figures 1 and 2 below summarize in a practical way the nutritional protocols in GVHD.
