# Supplementary material for: Brazilian Nutritional Consensus in Hematopoietic Stem Cell Transplantation: Graft- versus -host disease
Source: Einstein (Sao Paulo). 2020 Mar 13;18:eAE4799. doi: 10.31744/einstein_journal/2020AE4799 (PMC7069734; doi:10.31744/einstein_journal/2020AE4799)
Supplement: Translation

# Translation: Consenso Brasileiro de Nutrição no Transplante de Células Tronco Hematopoiéticas: doença do enxerto contra o hospedeiro

**Authors:** Andréa Z Pereira, Afonso Celso Vigorito, Alessandro de Moura Almeida, Alexandre de Almeida Candolo, Ana Carolina Leão Silva, Ana Elisa de Paula Brandão-Anjos, Bianca Laselva de Sá, Catarina Lôbo Santos de Souza, Cláudio Galvão de Castro, José Salvador Rodrigues de Oliveira, Juliana Bernardo Barban, Elaine Maria Borges Mancilha, Juliana Todaro, Lilian Pinheiro Lopes, Maria Cristina Martins de Almeida Macedo, Morgani Rodrigues, Paulo Cesar Ribeiro, Roberto Luiz da Silva, Telma Sigolo Roberto, Thays de Cássia Ruiz Rodrigues, Vergilio Antonio Rensi Colturato, Eduardo José de Alencar Paton, George Maurício Navarro Barros, Rosana Ducatti Souza Almeida, Maria Claudia Rodrigues Moreira, Mary Evelyn Flowers

**Affiliations:** 1 Brasil Hospital Israelita Albert Einstein, São Paulo, SP, Brasil .; 2 Brasil Universidade Estadual de Campinas, Campinas, SP, Brasil .; 3 Brasil Universidade Federal da Bahia, Salvador, BA, Brasil .; 4 Brasil Hospital de Câncer de Barretos, Barretos, SP, Brasil .; 5 Brasil Hospital Sírio-Libanês, São Paulo, SP, Brasil .; 6 Brasil Fundação Hospital Amaral Carvalho, Jaú, SP, Brasil .; 7 Brasil Complexo Hospitalar Universitário Professor Edgard Santos, Universidade Federal da Bahia, Salvador, BA, Brasil .; 8 Brasil Santa Casa de Misericórdia de Porto Alegre, Porto Alegre, RS, Brasil .; 9 Brasil Universidade Federal de São Paulo, São Paulo, SP, Brasil .; 10 Brasil Instituto Brasileiro de Controle do Câncer, São Paulo, SP, Brasil .; 11 Brasil Hospital São Luiz, São Paulo, SP, Brasil .; 12 Brasil Oncobio, Nova Lima, MG, Brasil .; 13 Brasil Instituto Nacional de Câncer José Alencar Gomes da Silva - INCA, Rio de Janeiro, RJ, Brasil .; 14 United States Seattle Cancer Care Alliance, Seattle, WA, United States .

**Keywords:** Nutrição, Doença enxerto-hospedeiro, Transplante de células-tronco hematopoéticas

## Abstract

O Consenso Brasileiro de Nutrição no Transplante de Células Tronco Hematopoiéticas: doença do enxerto contra o hospedeiro foi aprovado pela Sociedade Brasileira de Transplante de Medula Óssea, com a participação de 26 centros brasileiros de transplante de células-tronco hematopoiéticas. O Consenso descreve as principais condutas nutricionais em casos de doença do enxerto contra o hospedeiro, a principal complicação do transplante de células-tronco hematopoiéticas.

## TRANSPLANTE DE CÉULAS-TRONCO HEMATOPOIÉTICAS

Nos últimos 20 anos, as pesquisas desenvolvidas no transplante de céulas-tronco hematopoiéticas (TCTH) permitiram melhor seleção de doadores, redução na toxicidade advinda do condicionamento, com desenvolvimento de regimes de intensidade reduzida e melhora no tratamento de suporte, com a diminuição das complicações pós-transplante, aumentando, dessa forma, a sobrevida dos transplantados.^( 1 , 2 )^

A doença do enxerto contra o hospedeiro (DECH) é a maior causa de morbimortalidade relacionada ao TCTH alogênico, sendo responsável por grande impacto na qualidade de vida desses pacientes. Aproximadamente de 30% a 50% dos transpslantados alogênicos apresentam DECH no pós-TCTH.^( 3 )^A sobrevida global dos pacientes que apresentam DECH, particularmente a forma crônica, é de 72% em 1 ano e de 55% em 5 anos.^( 2 )^

## A FISIOPATOLOGIA DA DOENÇA DO ENXERTO CONTRA O HOSPEDEIRO

A DECH é causada pela ativação das células T que reconhecem os antígenos do hospedeiro como não próprios, provocando reação do tipo autoimune em órgãos do receptor, como pele, pulmões, fígado, trato gastrintestinal (TGI), timo, sistema hematopoiético e, possivelmente, mesmo o sistema nervoso central.^( 1 , 2 )^

A DECH aguda (DECH-a) grave se caracteriza por intensas lesões cutâneas, gastrintestinais e hepáticas, enquanto a forma crônica está associada a danos progressivos do tipo ulcerativos em mucosas e lesões sistêmicas em outros órgãos, como a pele e os pulmões.^( 3 )^

A DECH crônica (DECH-c) apresenta mais características de aloimunidade e imunodeficiência. Muito semelhante à DECH-a, a DECH-c também é induzida por células imunes do doador, mas sua fisiopatologia é menos compreendida. Apesar dos linfócitos T serem considerados o fator-chave em seu desenvolvimento, dados recentes revelam que as células B também apresentam importante papel.

Classicamente, o desenvolvimento da DECH pode ser dividido em três fases:^( 3 )^a primeira fase consiste na lesão dos tecidos do receptor pelos agentes utilizados nos regimes de condicionamento agressivos necessários para prevenir a recidiva das doenças neoplásicas e a rejeição do enxerto. Embora outros órgãos possam ser comprometidos em graus variados de gravidade, o sistema hematopoiético e o TGI são mais suscetíveis a esta toxicidade.

A segunda fase do desenvolvimento da DECH consiste na ativação de linfócitos T por células apresentadoras de antígenos do hospedeiro e, posteriormente, do doador, que adquirem funções de células T *helper* efetoras e secretam citocinas que, subsequentemente, aceleram a ativação imunológica.^( 4 , 5 )^

Na terceira fase da patogenia da DECH, a ativação imunológica de funções citotóxicas efetoras de células mediadoras, como as células T CD 81+, provoca lesões diretas das células-alvo características da DECH em órgãos como o fígado, a pele e o TGI.^( 6 , 7 )^

Em busca de maior conhecimento sobre a DECH e as maneiras de melhor controlá-la, no ano de 2005 foi estabelecido um consenso com a formação de um grupo de trabalho da *National Institutes of Health* (NIH), sendo definido que a apresentação clínica, não o tempo, é considerada o mais importante para o diagnóstico e para a diferenciação entre a DECH-c e a DECH-a. Alguns sinais e sintomas mostram-se similares em ambas; as diferenças, entretanto, são pungentes e permitem a delimitação de duas síndromes clínicas distintas.

## DOENÇA DO ENXERTO CONTRA O HOSPEDEIRO AGUDA

A DECH-a afeta primordialmente a pele, o fígado e o TGI. Na pele, são vistas lesões maculopapulares eritematosas coalescentes, que podem estar caracteristicamente na região plantar e na palma das mãos. O início da DECH hepática pode ocorrer pelo aumento das enzimas hepáticas e por sinais de colestase em exames laboratoriais. Os sintomas gastrintestinais mais inespecíficos são diarreia, náuseas e vômitos. Essa variedade de sintomas é amplamente diversa em gravidade.^( 2 - 4 )^

Os quadros podem ser extremamente agressivos, levando, por exemplo, à laceração da mucosa intestinal e sua eliminação fecal associada a hemorragias secundárias. Porém, muitas vezes, pode se apresentar com quadros leves, que necessitam de um diagnóstico diferencial invasivo e, muitas vezes, inconclusivo.^( 2 - 4 )^Por este motivo, a DECH-a foi estadiada ( Tabela 1 ) para se estabelecerem critérios de gravidade ( Tabela 2 ) e para padronizar uma maneira de avaliação em trabalhos acadêmicos universais.

**Tabela 1:** Estadiamento da doença do enxerto contra o hospedeiro aguda por órgão ( ^2^ - ^4^ )

Estágio	Achados cutâneos	Achados hepáticos	Achados intestinais
+	Exantema maculopapular em <25% da superfície corporal	Bilirrubina: 2-3mg/dL	Diarreia (500-1.000mL) persistente e náuseas
++	Exantema maculopapular em 25%-50% da superfície corporal	Bilirrubina: 3-6mg/dL	Diarreia (1.000-1.500mL)
+++	Eritrodermia generalizada	Bilirrubina: 6-15mg/dL	Diarreia >1.500mL)
++++	Descamação e bolhas	Bilirrubina: >15mg/dL	Dor com ou sem obstrução

**Tabela 2:** Graduação global da doença do enxerto contra o hospedeiro aguda ( ^2^ - ^4^ )

Grau/estágio	Pele	Fígado	Intestino	Distúrbio funcional
0 (nenhum)	0	0	0	0
I (leve)	+ a ++	0	0	0
II (moderado)	+ a +++	+	+	+
III (grave)	++ a +++	++ a +++	++ a +++	++
IV (com risco de morte)	++ a ++++	++ a ++++	++ a ++++	+++

## DOENÇA DO ENXERTO CONTRA O HOSPEDEIRO CRÔNICA

A DECH-c é uma síndrome clínico-patológica que envolve vários órgãos e sistemas, assemelhando-se muito às doenças autoimunes.

Esforços foram feitos no sentido de identificar os fatores de risco associados a um aumento da morbimortalidade nos pacientes com DECH-c. As variáveis identificadas incluíram envolvimento de múltiplos órgãos ou locais, piora da *performance status* , plaquetopenia no momento do diagnóstico, definida como contagem de plaquetas menor do que 100.000/µL, aparecimento progressivo da DECH-c, bilirrubinas elevadas e envolvimento extenso de pele (acometimento maior de 50% da superfície corpórea).^( 2 , 8 , 9 )^

Em 2005, o NIH desenvolveu um projeto para consenso dos critérios que devem ser utilizados em estudos clínicos da DECH-c.^( 10 , 11 )^Foram padronizadas as características utilizadas no diagnóstico, além de serem propostos meios para a pontuação dos órgãos envolvidos e avaliação global da gravidade.^( 8 , 12 )^

Estes critérios, revistos em 2014, são úteis para melhor análise da incidência da DECH-c, além de possibilitarem avaliar a gravidade do comprometimento de um órgão ou local, isolado ou combinado, e a influência na mortalidade relacionada ao transplante (MRT). Pelo consenso do NIH, sinais e sintomas diagnósticos se referem a manifestações que estabelecem a presença de DECH-c sem a necessidade de testes ou evidências de outros órgãos acometidos ( Tabela 3 ). Sinais e sintomas distintos se referem àquelas manifestações que não são comumente encontradas na DECH-c, mas são insuficientes para estabelecer diagnóstico preciso de DECH-c sem outros testes ou envolvimento de outros órgãos. Outras características definem manifestações raras, controversas e não específicas de DECH-c e não podem ser utilizadas para firmar o diagnóstico de DECH-c.^( 10 , 11 )^

**Tabela 3:** Sinais e sintomas relacionados à doença do enxerto contra o hospedeiro crônica ( ^10^ , ^11^ )

Órgão ou sítio	Diagnóstico (suficiente para estabelecer o diagnóstico de DECH-c)	Característico (presente na DECH, mas isoladamente é insuficiente para estabelecer o diagnóstico)	Outras características	Comuns à DECH-a e à DECH-c
Pele	Poiquiloderma	Despigmentação	Despigmentação	Eritema
	Líquen plano		Excesso ou falta de suor	*Rash* Maculopapular
	Alterações escleróticas		Ictose	Prurido
	Morfeia		Queratose pilar	
	Líquen esclerosante		Hipopigmentação	
			Hiperpigmentação	
Unha		Distrofia		
		Sulcos longitudinais		
		Onicolise		
		*Pterygium unguis*		
		Queda das unhas (geralmente simetricamente)		
Couro cabeludo e pelos		Alopecia total ou alopecia areata após a recuperação pós-quimioterapia	Afinamento dos pelos não explicável por outras causas	
		Lesões papuloescamosas	Pelos brancos precocemente	
Boca	Alterações tipo líquen	Xerostomia		Gengivite
	Placas hiperqueratóticas	Mucocele		Mucosite
	Restrição da abertura da boca por esclerose	Atrofia da mucosa		Eritema
		Pseudomembranas		Dor
		Úlceras		
Olho		Secura e dor nos olhos	Fotofobia	
		Conjuntivite cicatricial		
		Ceratoconjuntivite seca (teste de Schrimer <5mm/5 minutos)	Hiperpigmentação periorbital	
		Ceratopatia punctata em áreas confluentes	Blefarite	
Genitais	Líquen plano	Erosões		
	Estenose vaginal	Fissuras		
		Úlceras		
TGI	*Esophageal web*		Insuficiência pancreática exógena	Anorexia
	Constrição ou estenose no terço proximal do esôfago			Náusea
				Vômitos
				Diarreia
				Perda de peso
Fígado				Bilirrubina total e fosfatase alcalina >
				2 vezes acima do limite normal
				ALT ou AST >2 vezes limite superior
Pulmão	Bronquiolite obliterante diagnosticada com biópsia	Bronquiolite obliterante diagnosticada com Prova de função pulmonar ou Tomografia Computadorizada de Tórax		BOOP
Músculo, fascia, articulações	Fasciíte	Miosite ou polimiosite	Edema	
	Contraturas articulares secundárias à esclerose		Cãibras	
	Rigidez articular		Artralgia ou artrite	
Hematopoiético e imunológico				Trombocitopenia
				Eosinofilia
				Linfopenia
				Hipo ou hipergamaglobulinemia
				Autoanticorpos (AHAI e PTI)
Outros				Derrame pleural ou pericárdico
				Ascite
				Neuropatia periférica
				Síndrome nefrótica
				Miastenia grave
				Cardiomiopatia ou defeitos de condução cardíaca

DECH-c: doença do enxerto contra o hospedeiro crônica; DECH: doença do enxerto contra o hospedeiro; DECH-a: doença do enxerto contra o hospedeiro aguda; ALT: alanina aminotransferase; AST: aspartato aminotransferase; BOOP: Bronquiolite obliterante com pneumonia em organização; AHAI: anemia hemolítica autoimune; PTI: púrpura trombocitopênica imunológica.

O consenso recomenda os seguintes critérios para o diagnóstico da DECH-c:^( 8 , 9 )^distinção de DECH-a; presença de pelo menos um sinal clínico diagnóstico da DECH-c, ou presença de pelo menos uma manifestação distinta confirmada por biópsia pertinente de acordo com critérios histopatológicos definidos, testes laboratoriais, ou imagens radiológicas, no mesmo ou em outro órgão; e exclusão de outros diagnósticos possíveis.

A classificação revisada do NIH 2014 inclui oito órgãos como principais, por serem aqueles mais acometidos pela doença: pele, boca, olhos, TGI, fígado, pulmão, articulações e trato genital feminino. Os mais acometidos na DECH-c leve são pele, boca e fígado. Envolvimento do pulmão na DECH-c acrescenta por si só maior gravidade à doença, segundo o consenso, e, por isso, o dano pulmonar é um critério de gravidade considerado de grande importância nessa classificação.^( 8 , 9 )^

Para facilitar a graduação e estabelecer critérios padronizados de estágios da doença, os órgãos comumente afetados receberam pontuações e escalas de gravidade do dano produzido pela DECH-c. Cada órgão ou local recebeu pontuação de zero a 3, com zero representando nenhum envolvimento e 3, comprometimentos graves.^( 9 )^

A avaliação global da gravidade ( Tabela 4 ) nesse consenso é baseada no número de órgãos ou locais envolvidos e no grau de acometimento de cada órgão afetado. Os pacientes são considerados com diagnóstico de DECH-c leve quando apenas um ou dois órgãos (exceto os pulmões) forem afetados, sem dano funcional clinicamente significante, com pontuação máxima de 1 em todos os órgãos ou locais. O diagnóstico de DECH-c moderado é considerado quando pelo menos um órgão ou local apresentar comprometimento clínico significante, porém sem dano maior, com pontuação máxima de 2 em qualquer órgão ou local afetado, ou dois, três ou mais órgãos ou locais forem acometidos, porém sem prejuízo funcional clinicamente significante, com pontuação máxima de 1 em todos os órgãos ou locais afetados. Pontuação de 1 nos pulmões também é considerada moderada. DECH-c grave indica dano maior com a pontuação de 3 em qualquer órgão ou local. Pontuação ≥2 nos pulmões é considerada grave.^( 9 , 10 )^Estes valores são todos registrados em questionário validado pelo NIH e atualmente utilizado universalmente por inúmeros centros de pesquisa e assistência.

**Tabela 4:** Avaliação global de gravidade da doença do enxerto contra o hospedeiro crônica ( ^9^ , ^10^ )

Tipos DECH crônica	Critérios para classificação
DECH-c leve	1 ou 2 órgãos envolvidos +p ontuação nos órgãos envolvidos 1 + pontuação do pulmão 0
DECH-c moderada	3 ou mais órgãos envolvidos + pontuação 1 em cada órgão ou Pelo menos 1 órgão (exceto pulmão) com pontuação 2 ou Pontuação do pulmão 1
DECH-c grave	Pelo menos 1 órgão com pontuação 3 ou
	Pontuação do pulmão 2 ou 3
1. Na pele: a pontuação maior será usada para o cálculo da gravidade global	
2. No pulmão: VEF1 é usado no lugar da pontuação clínica para o cálculo da gravidade global	
3. Se uma anormalidade de um órgão é inequivocamente explicada por uma causa não associada à DECH, a pontuação deste órgão será zero para o cálculo da gravidade global	
4. Se uma anormalidade de um órgão é atribuída a causas multifatoriais (DECH mais outras causas), a pontuação do órgão será usada para o cálculo da gravidade global independentemente das causas contribuintes (a pontuação do órgão não será desconsiderada)	

DECH-c: doença do enxerto contra o hospedeiro crônica; DECH: doença do enxerto contra o hospedeiro; VEF: volume expiratório forçado.

O estudo da DECH-c inspira crescente interesse na comunidade acadêmica, agregado ao fato do estabelecimento recente de critérios que categorizaram a doença, baseados em evidências sedimentadas; foi iniciado o caminho para um maior entendimento da patogênese da DECH-c.

## INTRODUÇÃO SOBRE A IMPORTÂNCIA DO ESTADO NUTRICIONAL NA DOENÇA DO ENXERTO CONTRA O HOSPEDEIRO

Não existem dados de literatura claros sobre a interferência do estado nutricional prévio ao TCTH como causa de maior ou menor incidência de DECH, assim como sobre qual seria a melhor forma de realizar sua avaliação.^( 13 - 16 )^Alguns trabalhos relatam que taxas elevadas de desnutrição^( 17 )^e piora do estado nutricional associam-se a maior gravidade da DECH de TGI, de boca e de pulmão.^( 18 )^

Por outro lado, apesar da heterogeneidade dos estudos e de não se saber exatamente por meio de qual mecanismo se dá a interferência, tanto a obesidade quanto a desnutrição estão associadas a um maior risco para desenvolvimento de DECH.^( 19 , 20 )^

Recuperar ou melhorar o estado nutricional dos pacientes pré-TCHT poderia trazer consequências melhores ao desfecho.^( 20 )^

Entretanto, é conhecida a relação da DECH com os estados carenciais, como os défices vitamínicos.^( 14 - 16 )^

No período pós-transplante imediato (30 a 50 dias), as necessidades nutricionais refletem o aumento do aporte calórico-proteico em razão de condicionamento, infecções, DECH-a, febre e outras complicações metabólicas, afetando, principalmente, o balanço de proteínas, as necessidades energéticas e o metabolismo de micronutrientes.^( 21 , 22 )^

O estado nutricional na DECH-a ou na DECH-c é afetado por vários sintomas, que são amplamente discutidos posteriormente, como internações prolongadas e altas doses de corticosteroides, afetando profundamente a composição corporal com maior perda de massa muscular, retenção de líquido e aumento da gordura visceral, atingindo ainda mais o estado nutricional.^( 18 , 23 - 25 )^

Na DECH-c, nas manifestações orais, pulmonares e gastrintestinais, podemos encontrar até 29% de pacientes desnutridos em decorrência de dor na mucosa oral e atividade da doença, entre outros fatores.^( 18 )^Isso influi diretamente na redução da funcionalidade e da qualidade de vida dos pacientes.^( 18 )^

A DECH é uma condição complexa com efeitos significativos sobre a piora do estado nutricional, ocasionando redução da qualidade de vida e da funcionalidade dos pacientes.^( 18 , 25 )^Discutiremos, a seguir, tópicos específicos do estado e da terapia nutricional na DECH-a e na DECH-c.

## MICRO E MACRONUTRIENTES NA DOENÇA DO ENXERTO CONTRA O HOSPEDEIRO

A conduta dietoterápica depende da forma que a DECH se manifesta no paciente. A maioria dos pacientes inicia o tratamento com dieta relativamente saudável, mas rapidamente ocorre depleção dessa dieta. Isso se dá devido aos efeitos tóxicos diretos do tratamento ou às complicações secundárias, como as infecções e a própria DECH-a.^( 15 )^

A DECH moderada a grave e os regimes multifarmácias utilizados em sua prevenção e tratamento resultam em imunossupressão profunda e prolongada. Apesar dos avanços no manejo, a DECH continua problema significativo. Os pacientes frequentemente têm as necessidades nutricionais elevadas e alterações no metabolismo de carboidratos, gorduras e proteínas. Apresentam, ainda, dificuldade para comer por uma variedade de razões dependentes do envolvimento de órgãos e em geral requerem dietas modificadas, suplementos orais ou nutrição enteral (NE) ou parenteral (TNP) para prevenir a desnutrição.^( 26 )^

### Recomendações nutricionais: macronutrientes

#### Calorias

As necessidades nutricionais em pacientes submetidos ao TCTH aumentam devido ao catabolismo intenso.^( 27 )^É sugerido que as necessidades de energia durante a fase inicial do TCTH e da DECH sejam de até 130% a 150% do gasto estimado de energia basal, o que corresponde a 30 a 50kcal/kg de peso corporal por dia, e esse aumento da necessidade energética contribuiria para a perda ponderal desses pacientes.^( 25 , 28 , 29 )^Esse estado hipermetabólico crônico encontrado nesses pacientes é uma resposta às citocinas inflamatórias (fator de necrose tumoral alfa − TNF-α; interleucinas - IL − 1 e 6) e às alterações no níveis de norepinefrina e glucagon.^( 25 , 30 , 31 )^Estudos mostram aumento dos níveis séricos de glucagon ocasionando aumento de até 10% do metabolismo basal, principalmente pelo estímulo a gliconeogênese.^( 30 )^O aumento da norepinefrina nesses casos resulta em elevação da produção hepática de glicose, e também contribui para o aumento de metabolismo basal.^( 30 )^

Um estudo transversal com 13 pacientes comparou o requerimento energético por calorimetria indireta entre controles saudáveis e pacientes que apresentavam DECH-c extensa de pele, membranas mucocutâneas, pulmão, olhos e fígado, mostrando que houve pequeno aumento no requerimento de energia (1,9kcal/kg/dia ou 133kcal em uma pessoa de 70kg) e nas alterações das taxas de oxidação de gorduras e carboidratos.^( 30 )^

Além disso, foi demonstrado, em modelo animal, aumento da glicólise e do metabolismo de ácidos graxos para adequada função de células T alorreativas e indução da DECH.^( 32 , 33 )^Acredita-se, ainda, que o próprio tratamento da DECH possa ter efeitos no metabolismo energético dos pacientes, mas também são escassos os relatos sobre esse tópico.^( 22 )^

Nesse caso, recomendamos o uso de 30 a 50kcal/kg de peso corporal por dia para cálculo das necessidades calóricas nesses pacientes.

#### Proteínas e lipídios

A Organização Mundial de Saúde (OMS) recomenda como aceitável valor de ingestão proteica de 0,83g/kg de peso, sendo a capacidade máxima de síntese proteica atingida com ingestão de 1,5g/kg/dia.^( 25 )^Embora não existam estudos bem desenhados para embasarem tal recomendação, existe recomendação de manutenção de níveis mais elevados de ingestão proteica (cerca de 1,8 a 2,5g/kg/dia) nos casos de pacientes que desenvolveram DECH.^( 25 , 29 )^Tal recomendação baseia-se no fato da perda proteica por exsudação da mucosa intestinal e pelo efeito do uso crônico de corticoides no aumento da necessidade proteica.^( 29 , 34 - 40 )^

Os lipídios podem ser administrados com segurança como triacilgliceróis de cadeia longa (LCTs) ou LCT/mistura de triacilgliceróis de cadeia média, que geralmente contribuem com 30% a 40% de energia não proteica.^( 40 , 41 )^

#### Ômega 3

O ácido graxo ômega 3 desempenha papel como fator imunomodulador.^( 42 )^Foi teorizado que os lipídios poderiam modular vantajosamente a DECH, controlando a produção de citocinas por meio da via da prostaglandina E2. A manipulação de lipídio está associada ao controle da intolerância a glicose. Desse modo, temos aumento dos ácidos graxos monoinsaturados que substituiriam os ácidos graxos saturados ( Tabela 5 ).^( 42 )^

**Tabela 5:** Suplementos nutricionais recomendados para receptores de transplante de células-tronco hematopoiéticas com doença do enxerto contra o hospedeiro no *Baylor University Medical Center* ( ^29^ )

Suplementos	Motivo do consumo
Multivitamínico com minerais (com mínimo de ferro para o primeiro ano após o TCTH)	Para assegurar recursos adequados de vitaminas e minerais
	Metabolismo e anabolismo, especialmente se o paciente estiver com ingestão oral inadequada
Vitamina C (500mg/ 2vezes/dia)	Para ajudar na cicatrização de feridas
Zinco (22mg de sulfato de zinco/ 1 vez/ dia, durante 2 semanas)	Ajuda na cicatrização de feridas
	Para repor as quantidades perdidas na diarreia crônica
Ácido fólico (١mg/dia)	Para satisfazer as elevadas necessidades de produção de hemácias
	Certos medicamentos aumentam o metabolismo ou desperdício dessa vitamina e, portanto, precisa ser reposta
Cálcio com vitamina D (dose depende do nível sérico)*	Para minimizar a desmineralização óssea com uso de esteroides crônicos.
Ômega 3 (2 g/dia)	Interação com os níveis celulares para modificação das citocinas, reduzindo o processo inflamatório da DECH

* Nível sérico <10ng/mL-50.000UL/semana; 10-30ng/mL-10.000UL/semana. TCTH: Transplante de células-tronco hematopoiéticas; DECH: doença do enxerto contra o hospedeiro.

#### Glutamina

A utilização da glutamina é controversa. Parece haver alguns benefícios de seu uso oral na redução da mucosite e da DECH, enquanto a glutamina endovenosa pode reduzir as infecções.^( 43 )^

Segundo a revisão Cochrane, a glutamina não só modula a função do sistema imunológico no trato digestivo, mas também pode promover a cicatrização intestinal e reduzir a gravidade da mucosite e também da DECH-TGI.^( 44 )^Já a recente diretriz da *European Society for Clinical Nutrition and Metabolism* (ESPEN) conclui que não há evidências suficientes para recomendação da suplementação com glutamina para redução de toxicidade ao tratamento em pacientes com DECH-TGI.^( 36 )^

Então, devido a essa inconsistência da literatura, não se recomenda o uso de glutamina nessa população.

## Recomendações nutricionais: micronutrientes

### Vitamina B12

Os efeitos da DECH no estômago, reduzindo o fator intrínseco, e no intestino, reduzindo a absorção de vitamina B12, e o regime de condicionamento do TCTH, resultando na degeneração das células de cripta, associam-se à diminuição dessa vitamina ( Tabela 5 ).^( 25 )^

### Vitamina C

Estudos mostram que a vitamina C tem importante papel no combate de mucosite em pacientes com DECH. Pacientes com deficiência em vitamina C que receberam tratamento com 2.000mg/mês de ácido ascórbico apresentaram significantes melhorias visuais na mucosite e conseguiram voltar a comer^( 45 )^( Tabela 5 ).

### Zinco

A diarreia crônica e a malabsorção ocasionadas pela DECH podem levar a uma deficiência de zinco, que é importante na manutenção do paladar e da integridade da mucosa gastrintestinal.^( 25 )^Além disso, o zinco atua na cicatrização e na percepção do sabor, e é importante defesa contra as infecções intestinais, devido à manutenção da integridade da mucosa intestinal.^( 46 )^

Diversos estudos recomendam sua suplementação em pacientes com DECH, como Roberts et al.,^( 29 )^que recomendam que a suplementação de zinco é interessante para o tratamento de lesões recorrentes. Ripamonti et al.,^( 47 )^sugerem que a suplementação (até 3 doses/dia de 45mg ZnSO_4_) é segura e efetiva para tratamento da percepção do sabor.

Além disso, estudos experimentais têm sugerido papel desse elemento na ativação de células T regulatórias, podendo ser interessante para o TCTH^( 48 )^( Tabela 5 ).

### Vitamina D

Alguns estudos já descreveram a presença de deficiência de vitamina D em pacientes após o TCTH e sua relação com o desenvolvimento da DECH e com a redução da densidade mineral óssea.^( 49 )^A despeito de sua associação com uma nutrição inadequada, a deficiência de vitamina D não tem sido caracterizada como complicação direta do desenvolvimento da DECH,^( 50 )^mas parece ter papel em seu desenvolvimento. Sproat et al., em estudo retrospectivo com número reduzido de pacientes (58 pacientes transplantados entre 2000 e 2009), relataram prevalência de hipovitaminose D de 89,7%, e a maioria destes pacientes tiveram DECH (94,8%) e fizeram uso de corticosteroides (98,3%).^( 51 )^Contudo, outros estudos também observaram essa associação de baixos níveis séricos de vitamina D (<25ng/mL) e DECH, além de reativação de citomegalovírus (CMV) pós-transplante.^( 52 , 53 )^

A redução dos efeitos da DECH pode ser explicada pelo aparente papel da vitamina D no sistema imunológico, regulando o funcionamento de células dendríticas, macrófagos e linfócitos B e T.^( 54 - 56 )^

Os pacientes com DECH-a tratados com corticosteroides revelam tendência para maior diminuição da vitamina D. O monitoramento de seus níveis e, se necessário, o tratamento para sua correção, podem ser indicados em intervalos regulares antes do TCTH e durante o seguimento destes pacientes.^( 49 )^

A reposição de cálcio e vitamina D em combinação com bifosfonatos, ou a suplementação com metabólitos ativos como 1,25 (OH)_2_D3 vitamina D ou 25 (OH)_3_vitamina D trazem efeitos benéficos para a massa óssea e a modulação da DECH.^( 57 , 58 )^

O estudo da suplementação de vitamina D no TCTH é relativamente recente, mas já oferece resultados promissores. A dosagem de seu nível sérico deveria ser realizada no pré-TCTH e no pós-TCTH, e a deficiência deve ser corrigida.

### Magnésio

A principal alteração do metabolismo nos pacientes com DECH é a hipomagnesemia, causada pelos inibidores de calcineurina, uma das classes de medicações mais utilizadas, tanto na profilaxia quanto no tratamento da doença. Contudo, há relatos de caso em que houve hipermagnesemia severa após o uso de medicações laxantes, com alta concentração de magnésio, provavelmente associadas à desidratação e à alta permeabilidade intestinal vista na DECH.^( 59 )^

### Ferro

A sobrecarga de ferro é uma complicação comum dos TCTH, devido ao aumento de absorção de ferro secundária à anemia e às múltiplas transfusões. A sobrecarga de ferro pode aumentar o risco de DECH, sobretudo aguda, pela tendência a causar toxicidade hepática direta. Além disso, a ferritina parece ser marcador de pior prognóstico em pacientes com DECH.^( 25 , 60 )^Recomenda-se o uso de multivitamínicos que não contenham ferro nessa população.^( 61 )^

As recomendações nutricionais em pacientes com doença do enxerto contra o hospedeiro são apresentadas no tabela 6 .

**Tabela 6:** Recomendações nutricionais em pacientes com doença do enxerto contra o hospedeiro

Avaliar o estado nutricional por especialista
Manter as necessidades calóricas em 30 a 50kcal/kg e proteicas em 1,5-2g/kg
Monitorar o peso e a ingestão de nutrientes no primeiro ano após o transplante; pacientes com DECH ativa necessitam de monitoramento mais longo
Aconselhar e monitorar o suporte nutricional especificamente para os pacientes com DECH do trato gastrintestinal; iniciar o apoio nutricional especializado em pacientes com disfunção significativa do trato gastrintestinal e anorexia, que são incapazes de manter o peso corporal adequado
Suplementar com multivitamínicos/minerais (sem ferro, devido ao risco de hemocromatose); outros suplementos, como vitamina C, zinco, ácido fólico e ômega 3, podem ser benéficos
Aconselhar aspectos nutricionais referentes à segurança alimentar e orientar o risco de transmissão de doenças por alimentos durante a imunossupressão

Fonte: Adaptado de Roberts S, Thompson J. Graft-vs-host disease: nutrition therapy in a challenging condition. Nutr Clin Pract. 2005;20(4):440-50.^(^^29^^)^DECH: com doença do enxerto contra o hospedeiro.

## COMPLICAÇÕES NUTRICIONAIS MAIS COMUNS NA DOENÇA DO ENXERTO CONTRA O HOSPEDEIRO

Devido à importância desse tema, tentaremos revisar a seguir as principais complicações nutricionais da DECH, tanto causadas por seu desenvolvimento quanto relacionadas à sua terapia. Os efeitos colaterais relacionados a aspectos nutricionais das principais medicações utilizadas para tratamento da DECH estão demonstrados na tabela 7 .

**Tabela 7:** Principais medicações e terapias imunossupressoras utilizadas no tratamento da doença do enxerto contra o hospedeiro e seus efeitos colaterais nutricionais e metabólicos

Medicação/terapia	Mecanismo de ação	Efeitos nutricionais e metabólicos
Corticoides	Resposta anti-inflamatória, inibe IL-1, diminui IL-2 e suprime a proliferação de linfócitos	Retenção de sódio e água, hiperglicemia, hipercolesterolemia, aumento de apetite, ganho de peso, desmineralização óssea e efeitos musculares
Ciclosporina/tacrolimus	Inibe proliferação/resposta de linfócitos T e altera produção de IL-2	Hipertensão, dislipidemia, hiperglicemia, hipomagnesemia, hipercalemia, nefrotoxicidade, neurotoxicidade, náuseas, vômitos, alterações do paladar e diarreia
Metotrexato	Antimetabólico e imunossupressor	Anorexia, náuseas, vômitos, diarreia, estomatite, mucosite, hepatotoxicidade e nefrotoxicidade
Micofenolato de mofetila	Diminui a ativação linfocítica e a proliferação de células B e T; suprime a formação de anticorpos	Náuseas, vômitos, diarreia, constipação, sangramento gastrintestinal e edema periférico
Sirolimus	Inibe a proliferação de linfócitos B e T	Dislipidemia, hipertensão e edema periférico
Talidomida	Propriedades imunossupressoras e anti-inflamatórias	Neuropatia e constipação
Globulina antitimócito (ATG)	Diminuição dos linfócitos circulantes	Dor abdominal, náuseas, vômitos, diarreia, hipercalemia, hipertensão e edema periférico
Etarnecept	Antagonista do TNF-α	Dor abdominal e vômitos
Ácido ursodesoxicólico	Substitui ácidos biliares nativos humanos; diminui a expressão de antígenos de HLA nos hepatócitos	Náuseas, vômitos, diarreia e dor abdominal
Daclizumabe	Anticorpo anti-IL-2	Vômitos, edema, hipertensão e hipotensão
Azatioprina	Previne a proliferação citotóxica de linfócitos T e B por inibir a síntese de DNA e RNA	Hipersensibilidade gastrintestinal, hepatotoxicidade, anemia megaloblástica e pancreatite
Hidroxicloroquina	Interfere no processamento de antígenos e na apresentação, proliferação, produção de TNF-α e citotoxicidade	Náuseas, vômitos e diarreia
Infliximabe	Anticorpo anti-TNF-alfa.	Dor abdominal, náuseas, vômitos
Psoralen e PUVA	Interfere na apresentação de antígenos e na produção de citocinas pró-inflamatórias	Náuseas e hepatotoxicidade
Fotoaférese extracorpórea	Induz apoptose de células T alorreativas, fotoinativação de células apresentadoras de antígenos	Hipocalcemia (uso de citrato) e transtornos gastrintestinais
Ciclofosfamida	Atividade imunossupressora e bloqueio de crescimento celular por ligação de metabólitos ao DNA	Anorexia, náuseas, vômitos e mucosite
Rituximabe	Anticorpo anti-CD20	Dor abdominal, diarreia, náuseas, vômitos, hipertensão e hiperglicemia
Pentostatin	Bloqueio da síntese de DNA	Náuseas, vômitos, fadiga, diarreia, anorexia e estomatite
Imatinibe	Inibição do PDGF-r	Náuseas, fadiga, diarreia, dor abdominal, vômito, ganho de peso, hepatotoxicidade, hiperglicemia e miopatia

Fonte: Adaptado de Roberts S, Thompson J. Clinical Observations Graft- vs -Host Disease : Nutrition Therapy in a Challenging Condition. Nutr Clin Pract. 2005;20:440-50.^(^^29^^)^IL: interleucina; TNF-α: fator de necrose tumoral alfa; HLA: antígeno humano leucocitário; PUVA: psolaren + irradiação ultravioleta A; PDGF-r: receptor do fator de crescimento derivado de plaquetas.

## ALTERAÇÕES MECÂNICAS NO TRATO GASTRINTESTINAL

O TGI é envolvido na maioria dos pacientes com DECH, podendo acometer qualquer parte do tubo digestivo. Embora sejam mais raras, as alterações mecânicas e estruturais do trato digestivo merecem ser relatadas, devido à sua gravidade e à necessidade de abordagem precoce.^( 62 )^

As complicações esofágicas são raras e incluem ulceração, varizes esofágicas e doença vesiculoide. As disfagias com severa estenose requerem dilatação esofágica.^( 62 )^

Uma das complicações mais graves do intestino é a perfuração intestinal; no entanto, a mais frequente é a diarreia.^( 63 )^

## ALTERAÇÃO NA ABSORÇÃO DE NUTRIENTES

A alteração da absorção vista em pacientes com DECH pode estar associada a alterações hepáticas e pancreáticas. As alterações hepáticas podem ser decorrentes da excreção prejudicada de sais biliares, tendo importante papel no metabolismo lipídico.^( 64 )^

As alterações pancreáticas já foram relatadas em autópsias de modelos experimentais, sendo associadas por acometimento da DECH; contudo, estas alterações, que podem incluir atrofia, também podem ser decorrentes de medicações como azatioprina, ciclosporina e corticoides.^( 25 )^Os principais sintomas da insuficiência exócrina pancreática são esteatorreia, fadiga, dor abdominal, perda ponderal e flatulência. Tais sintomas estão mais frequentes no pós-transplante em pacientes com sinais de DECH, sendo mais frequentes entre graus mais avançados da doença.^( 65 , 66 )^

Além da função pancreática, a DECH em intestino delgado também tem sido estudada como possível causa de alteração digestiva em pacientes pós-transplante. Além dos estudos endoscópicos por cápsula, um marcador que vem sendo testado é a citrulina. O intestino delgado é a principal fonte deste aminoácido em nosso organismo. Estudos prévios em pacientes sem DECH demonstraram correlação entre os níveis plasmáticos reduzidos de citrulina e dano intestinal.^( 67 , 68 )^Tais achados foram também descritos entre pacientes com DECH intestinal.^( 69 )^Esse aminoácido tem demonstrado ser promissor, ainda, na predição do desenvolvimento de DECH,^( 70 )^embora necessite de mais dados na literatura para seu uso na prática clínica.

## DIARREIA E ENTEROPATIA PERDEDORA DE PROTEÍNAS

A diarreia é um dos principais sintomas da DECH de trato digestivo baixo. Contudo, sua etiologia nesta entidade é multifatorial, podendo incluir atrofia vilosa, ulceração de mucosa, disfunção secretória, fatores osmóticos, insuficiência pancreática e alteração do trânsito intestinal. Ela é frequentemente esverdeada, líquida, mucoide e pode ser de grande quantidade.^( 25 , 71 )^

O dano do tecido gastrintestinal provocado pela DECH pode levar a diversos problemas, incluindo desidratação, perda de eletrólitos e enteropatia perdedora de proteínas. Tal situação é definida pelo aumento da alfa 1-antitripsina (>2,2mg/g de peso fecal seco) em amostras fecais e ocorre, especialmente, em pacientes com DECH de trato digestivo.^( 25 , 35 )^

Papadopoulou et al., estudaram amostra de 47 pacientes submetidos ao TCTH, sendo 42 destes alogênicos. Eles encontraram que 91% dos episódios de diarreia estavam associados à enteropatia perdedora de proteína, e a quantidade de proteína perdida era mais severa entre pacientes com DECH (19,4mg/g) do que entre pacientes com outras causas de diarreia, como rotavírus, infecção por CMV ou causas incertas (6,7mg/g).^( 35 )^

A quantidade de perda proteica também parece estar correlacionada com a gravidade da DECH, principalmente entre pacientes submetidos a condicionamentos mieloablativos.^( 72 )^Além disso, os pacientes com DECH costumam apresentar elevação persistente da quantidade de proteína perdida nas fezes, diferentemente do que acontece com indivíduos com outros transtornos diarreicos.^( 34 )^

## EFEITOS SOBRE APETITE

Além dos efeitos do regime de condicionamento e das medicações imunossupressoras e de suporte utilizadas, o desenvolvimento da DECH pode, por si só, ter efeito sobre o apetite. Malone et al., demonstraram maior ingestão oral entre pacientes sem DECH ou com DECH-a grau 1 comparado com os demais.^( 73 )^Os sintomas associados à DECH, sobretudo do trato digestivo, são relacionados como agentes causais da nutrição inadequada. No entanto, isso não é tão facilmente explicado. Parece que a própria atividade da DECH pode ter papel sobre a supressão do apetite.^( 18 )^

## ALTERAÇÕES NO METABOLISMO DE CARBOIDRATO E LIPÍDIOS

O controle glicêmico é importante durante o período pós-transplante. Hiperglicemia causa não somente impacto na função imune, mas também causa prejuízos em outros tecidos, como disfunção endotelial, elevação das citocinas pró-inflamatorias, catabolismos muscular e adiposo. Teoricamente, a hiperglicemia pode aumentar o nível de citocinas e o risco de doenças infecciosas, o que pode levar a um risco aumentado de DECH. Por outro lado, a DECH pode também, por meio de mecanismos inflamatórios, levar a um estado de hiperglicemia.^( 74 )^Além disso, os corticosteroides utilizados no tratamento da DECH possuem hiperglicemia como um dos efeitos colaterais mais comuns.^( 29 )^

Com relação à dislipidemia, diversas medicações utilizadas no tratamento da DECH estão relacionadas ao desenvolvimento desta complicação ( Tabela 7 ). Contudo, não apenas as medicações imunossupressoras afetam a homestase lipídica. A DECH do fígado pode levar a elevações de colesterol e triglicerídeos, pela inabilidade dos sais biliares e colesterol serem excretados pelo duto biliar.^( 64 )^Ademais, a síndrome nefrótica, que pode ser uma complicação grave da DECH, também pode levar à dislipidemia significante.^( 64 )^

## PERDA DE MASSA MAGRA E MIOPATIA

A perda de massa magra é frequente entre pacientes com DECH e consequência das alterações nutricionais por ela ocasionadas. A terapia com corticosteroide influencia de forma significativa nessa complicação. O desenvolvimento de DECH-c parece ser fator de risco independente para a perda de massa corpórea magra, sendo maior a probabilidade entre aqueles com DECH extensa e naqueles que necessitaram de uso de corticosteroides.^( 75 , 76 )^

## INTERVENÇÃO NUTRICIONAL NA DOENÇA DO ENXERTO CONTRA O HOSPEDEIRO AGUDA E CRÔNICA

Os pacientes com DECH têm dificuldade para ingerir alimentos por várias razões, dependendo do órgão envolvido. Frequentemente, necessitam de modificações na dieta, suplementos orais e terapia de suporte nutricional (TSN), para prevenir ou tratar a desnutrição.^( 77 )^

Segundo Bassim et al.,^( 18 )^as principais indicações para o início da TSN são náuseas e vômitos não controlados, diarreia volumosa, dor na mucosa oral e esofágica, disfagia, disgeusia, xerostomia, anorexia, saciedade precoce e perda de peso. Em especial, a DECH-a de TGI e a DECH-c oral, gastrintestinal e pulmonar produzem desnutrição grave e levam a um comprometimento da capacidade funcional e da qualidade de vida dos pacientes, daí a necessidade do início precoce de TSN.

A terapia nutricional é de extrema importância como suporte ao tratamento para combater os efeitos deletérios da DECH e contornar os efeitos adversos dos medicamentos.^( 15 , 25 , 77 , 78 )^

## MANEJO DE SINTOMAS POR MODIFICAÇÕES NUTRICIONAIS

De acordo com o Consenso de Nutrição Oncológica do Instituto Nacional do Câncer José de Alencar,^( 21 )^algumas intervenções nutricionais podem ser orientadas para melhorar e controlar os sintomas gastrintestinais.

### Saciedade precoce

Conscientizar o paciente da importância da alimentação; realizar fracionamento da dieta (de seis a oito refeições/dia); modificar as fibras da dieta por meio de cocção e/ou trituração, para reduzir a saciedade (frutas sem casca, legumes e verduras cozidos, sopas e sucos liquidificados); aumentar a densidade calórica e proteica das refeições; não ingerir líquidos durante as refeições; utilizar carnes magras, cozidas, picadas, desfiadas ou moídas em porções reduzidas; evitar alimentos e preparações ricos em gorduras; e preferir bebidas não gaseificadas.^( 21 )^

### Diarreia

O fracionamento da dieta é importante, bem como reduzir o volume de alimentos por refeição; avaliar a restrição de lactose, sacarose, glúten, gordura, fibras insolúveis, cafeína e teína; aumentar a ingestão hídrica e de líquidos isotônicos para, no mínimo, 3L/dia; evitar alimentos flatulentos e hiperosmolares; e evitar temperaturas extremas.^( 21 )^

### Disfagia

Acompanhamento com o fonoaudiólogo, para adequada modificação da dieta; orientar o paciente quanto a cuidados com alimentos secos e duros, e preferir alimentos pastosos, de fácil mastigação e deglutição; ingerir pequenos volumes de líquidos junto às refeições, para facilitar a mastigação e a deglutição; e manter a cabeceira elevada para alimentar-se.^( 21 )^

### Xerostomia

O consumo de, no mínimo, 2L/dia de água e líquidos em geral até 3L/dia é necessário; estimular a ingestão de alimentos mais prazerosos; adequar a consistência dos alimentos conforme a aceitação do paciente; evitar o consumo de café, chá e refrigerantes que contenham cafeína; manter higiene oral e hidratação labial; utilizar gotas de limão nas saladas e bebidas; se necessário, ingerir líquidos junto às refeições, para facilitar a mastigação e a deglutição; uso de balas cítricas e mentoladas sem açúcar; temperar os alimentos com ervas aromáticas, evitando sal e condimentos em excesso; mastigar e chupar gelo feito de água, água de coco e suco de frutas ou picolés.^( 21 )^

### Náuseas e vômitos

É necessário orientar uma alimentação fracionada em pequenos volumes; dar preferência a alimentos mais secos, cítricos, salgados e frios ou gelados; manter a higiene oral; não realizar jejuns prolongados; chupar gelo 40 minutos antes das refeições; não comer frituras e alimentos gordurosos; evitar alimentos e preparações que exalem odor forte e de sabor muito doce; procurar realizar as refeições em locais arejados; não ingerir líquidos durante as refeições, utilizando-os em pequenas quantidades nos intervalos, preferencialmente gelados (por exemplo, picolé); não deitar logo após as refeições; e utilizar gengibre pelo seu efeito antiemético, por meio de infusão, como tempero ou adicionado a sucos.^( 21 )^

### Anorexia

O paciente deve ser orientado acerca da importância de uma ingestão alimentar adequada; fracionamento da dieta e volume reduzido; maior densidade calórica e proteica das refeições; consumir alimentos com melhor tolerância e consistência, conforme preferências do paciente.^( 21 )^

### Odinofagia

A consistência da dieta deve ser modificada conforme a tolerância; melhorar a densidade calórica e proteica das refeições; boa higiene oral; não consumir alimentos secos, duros, cítricos, salgados, picantes e condimentados; evitar temperaturas extremas.^( 21 )^

### Dieta oral

Nos envolvimentos leves da cavidade oral, deve-se evitar o consumo de alimentos ácidos; em casos mais graves com estenose de esôfago, a consistência e a temperatura da dieta devem ser modificadas, dando preferência para forma líquida ou liquidificada, servidas em temperatura moderada ou ambiente.^( 21 , 74 )^

Durante o tratamento com altas doses de glicocorticoides e/ou inibidores de calcineurina é importante a orientação adequada ao paciente. São recomendadas refeições frequentes e fracionadas, alimentação rica em fibras solúveis e insolúveis, dieta hiperproteica, com redução de carboidratos simples e de alto índice glicêmico, redução de sódio, boa ingestão hídrica e consumo adequado de alimentos-fontes de vitamina D, cálcio, magnésio, zinco e, se necessário, a suplementação destes elementos.^( 79 , 80 )^

### Suplementos orais

Independentemente do tipo e do grau da DECH, quando o paciente apresenta ingestão alimentar abaixo de 70% das necessidades energéticas nos últimos 3 dias e sintomas que prejudiquem a alimentação adequada é importante intervir com o uso de suplementos nutricionais hipercalóricos e hiperproteicos (adaptados de acordo com a liberação e a fase da dieta restrita, no caso de DECH intestinal). A suspensão do suplemento nutricional via oral é indicada somente na presença de instabilidade hemodinâmica, esofagite ou mucosite grave que impeçam a ingestão oral adequada, obstrução do TGI, vômitos incoercíveis, risco de broncoaspiração, recusa do paciente e intolerância ao suplemento.^( 21 )^

## NUTRIÇÃO ENTERAL

Caso a ingestão alimentar esteja abaixo de 60% das necessidades energéticas nos últimos 3 dias ou a via oral estiver contraindicada, a NE pode ser prescrita.^( 21 )^A via enteral, se tolerável e clinicamente possível, pode ser escolhida por manter a função digestiva e a integridade da barreira mucosa, prevenindo a translocação bacteriana no trato digestivo.^( 25 )^

De acordo com a *American Society for Parental and Enteral Nutrition* ,^( 77 )^quando a contagem de neutrófilos e a de plaquetas estão dentro da normalidade e o TGI está cicatrizado, a NE é segura para transição da terapia nutricional parenteral para dieta oral ou quando há necessidade de TSN no caso de DECH, entre outras complicações tardias do TCTH.

Segundo revisão sistemática realizada por Baumgartner et al.,^( 15 )^vários estudos têm comparado a NE com TNP, mostrando resultados superiores para a via enteral e moderada a alta tolerância à sonda, sendo a TNP recomendada somente em casos de insuficiência gastrintestinal. A NE é contraindicada na presença de instabilidade hemodinâmica e/ou piora da dor abdominal, distensão abdominal, mucosite, diarreia, vômitos incoercíveis, íleo paralítico e sangramento intestinal.^( 21 )^

Existem fortes evidências indicando que a introdução precoce de NE pode diminuir tanto a incidência quanto a gravidade da DECH-TGI, podendo ser uma forma de profilaxia. Além disso, a NE está associada a uma menor mortalidade relacionada a infecções e a períodos mais curtos de enxertia de neutrófilos.^( 81 )^

## DIETA PARENTERAL

A TNP também pode ser indicada para pacientes que apresentam aceitação da dieta oral inferior a 60% a 70% das necessidades nutricionais por 3 dias consecutivos,^( 82 )^ou, ainda, em pacientes com défice energético-proteico, com uso exclusivo de NE.^( 83 )^

As diretrizes da ESPEN recomendam dieta oral ou enteral, desde que sejam possíveis, mas, no caso de vômitos, diarreia incoercível, mucosite grave ou má absorção importante, a TNP deve ser a via preferencial.^( 36 )^

Estudos demonstram que pacientes com DECH graus III-IV recebem mais TNP que pacientes com DECH graus I-II, não estando isentos de apresentarem complicações clínicas, relacionadas com a progressão do número de dias que recebem a TNP.^( 84 )^

É importante salientar alguns cuidados com a prescrição e o acompanhamento da TNP. Pacientes desnutridos, com risco de síndrome de realimentação, devem receber aporte energético progressivo, na fase inicial (primeiro ao terceiro dia), com 20% das necessidades energéticas basais. A oferta proteica pode ser oferecida desde o início, respeitando as funções renais e hepáticas. O controle glicêmico deve ser realizado respeitando níveis de glicemia menores que 180mg/dL e com a prevenção de hipertrigliceridemia, mantendo nível sérico inferior a 400mg/dL.^( 83 )^Além do acompanhamento da função hepática, com dosagem de AST, ALT, gama glutamil transferase, fosfatase alcalina e bilirrubina duas vezes por semana, devem ser incluídos nos exames de rotina ureia, creatinina, dosagem sérica de eletrólitos (potássio, magnésio, fósforo, cálcio e sódio), colesterol total e frações. O desmame da TNP deve ser gradativo, respeitando a oferta e a aceitação da dieta oral ou enteral pelo paciente.^( 83 )^

### Manejo nutricional na doença do enxerto contra o hospedeiro intestinal

A avaliação nutricional dos pacientes acometidos por essa complicação pode ser muito difícil, visto que muitos apresentam retenção de líquidos relacionados a baixos níveis de albumina sérica, o que mascara a perda de peso corporal. Além disso, o tratamento padrão da DECH-TGI é corticoterapia, que tem efeitos diretos na composição corporal, levando a aumento da gordura corporal, diminuição da massa magra, retenção hídrica e de sódio, hipertrigliceridemia, hipercolesterolemia, sarcopenia e desmineralização óssea, podendo mascarar o estado nutricional dos pacientes.^( 25 )^

Os objetivos da terapia nutricional na DECH-TGI são fornecer o suporte nutricional adequado e individualizado para manter ou recuperar o estado nutricional do paciente, controlar os sintomas, reestabelecer a integridade da mucosa intestinal, satisfazer o paciente e lhe promover qualidade de vida, sempre que possível.^( 25 , 85 )^

## TERAPIA NUTRICIONAL NA DOENÇA DO ENXERTO CONTRA O HOSPEDEIRO INTESTINAL

### Dieta oral

No *National Cancer Center Japan* , realizou-se um estudo com a terapia nutricional escalonada com protocolo próprio, tendo sido observado que o estado nutricional dos pacientes tende a melhorar com esse tipo de terapia; porém, não foi observada melhora na evolução do grau da DECH-TGI.^( 86 )^

No *Seattle Cancer Care Alliance* , de acordo com o guia para médicos, a terapia nutricional também é baseada nesse tipo de terapia nutricional escalonada, e a evolução da dieta do paciente ocorre de acordo com sua tolerância e sintomas apresentados.^( 87 )^

O uso de suplementos orais artesanais ou industrializados pode se dar a partir da etapa 2, quando a ingestão alimentar não atingir as necessidades nutricionais recomendadas, devendo seguir as mesmas características da dieta da etapa correspondente e a vontade do paciente.

Baseado nessa literatura segue a tabela 8 , na qual a terapia nutricional do paciente deve alterada de acordo com os sintomas, a evolução clínica e sua tolerância; se, ao passar para uma etapa subsequente, o paciente não tolerar a alteração da dieta, esta deve ser regredida para a etapa anterior.

**Tabela 8:** Progressão escalonada da terapia nutricional de pacientes acometidos por doença do enxerto contra o hospedeiro intestinal

Etapa	Sintomas	Terapia nutricional
1. Repouso intestinal	Grande volume de diarreia aquosa (acima de 1.000mL/dia); cólicas intestinais; depleção da albumina sérica; diminuição do trânsito intestinal; obstrução intestinal; náuseas e vômito	Somente TNP
2. Introdução de alimentação oral/enteral	Volume de diarreia menor que 500mL/dia; diminuição das cólicas intestinais; melhora do tempo de trânsito intestinal; diminuição das náuseas e vômitos	TNP + dieta oral/enteral com características: líquida isosmótica, sem resíduos, sem lactose, sem ácidos e hipogordurosa
3. Introdução de alimentos sólidos	Ausência ou diminuição das cólicas e fezes mais consistentes	Dieta oral/enteral com características: alimentos sólidos, sem resíduos, sem lactose, hipogordurosa e sem ácidos e irritantes gástricos
4. Expansão da dieta	Ausência ou diminuição das cólicas e fezes mais consistentes	Dieta oral/enteral (se necessário, de acordo com a individualidade do paciente) com características: pobre em fibras, lactose, ácidos, irritantes gástricos e gordura de acordo com tolerância do paciente
5. Introdução da dieta habitual do paciente	Ausência de cólicas e fezes em consistência normal	Dieta oral com características: introdução gradual e de acordo com a tolerância do paciente de alimentos ácidos, irritantes gástricos, com fibras, lactose e gordura

Fonte: Adaptado de Fred Hutchinson Cancer Research Center. Long-term follow-up after hematopoietic stem cell transplant. Fred Hutchinson Cancer Research Center/ Seattle Cancer Care Alliance [Internet]. Seattle, WA; 2014 [citado 25 Jun 2019]. Disponível em: https://www.fredhutch.org/content/dam/public/Treatment-Suport/Long-Term-Follow-Up/physician.pdf;^(^^80^^)^Gauvreau JM, Lenssen P, Cheney CL, Aker SN, Hutchinson ML, Barale KV. Nutritional management of patients with intestinal graft- *versus* -host disease. J Am Diet Assoc. 1981;79(6):673-7.^(^^88^^)^TNP: terapia de nutrição parenteral.

### Dieta parenteral

Os pacientes com DECH-TGI na fase aguda e inicial da doença geralmente apresentam volume de diarreia >1.000mL/dia, inviabilizando a nutrição por via oral ou enteral, suficiente para suprir suas necessidades nutricionais mínimas. Isso pode durar dias ou semanas. Desse modo, a terapia nutricional mais indicada seria o repouso do TGI com jejum e uso da terapia nutricional por via parenteral.^( 28 )^

A abordagem mais tradicional no manejo nutricional na DECH-TGI tem sido a administração de TNP e o início da ingestão oral após a recuperação dos sintomas do TGI. Porém, devido ao tempo prolongado de uso da TNP, ocorrem danos à mucosa intestinal, induzindo a atrofia e mais disfunções intestinais.

### Dieta enteral

A introdução da dieta oral ou NE a partir da etapa 2 deve acontecer após redução do volume de diarreia para menos que 500mL/dia; diminuição das cólicas intestinais; melhora do tempo de trânsito intestinal; diminuição das náuseas e vômitos. Essa introdução gradativa deve ser priorizada, pois auxilia na manutenção do trofismo intestinal, ajudando a preservar a barreira mucosa, e a imunidade local e sistêmica, e, também, a evitar translocação bacteriana.^( 13 )^

A escolha pela via oral, enteral ou concomitante durante as etapas de evolução da terapia nutricional se dá de acordo com os sintomas, a possibilidade de alimentação por via oral e nas situações em que a dieta oral for inadequada para alcançar as necessidades nutricionais.

A NE, se escolhida como via de nutrição ou de suplementação, deve seguir as características de cada etapa. A partir da etapa 2, o paciente não tolera grandes volumes de dieta oral e/ou enteral, de forma que a TNP não precisa ser suspensa, a fim de suprir todas as necessidades nutricionais do paciente.

Estudos mostram que o uso de NE na DECH-TGI, em comparação ao uso da TNP, por preservar o trofismo intestinal, melhorar a função da barreira do intestino e, assim, diminuir o risco de translocação bacteriana, reduz as complicações infecciosas.^( 89 , 90 )^Porém, historicamente, os centros de transplante preferem a TNP à NE, dificultando seu uso precoce ou durante o TCTH.

## A IMPORTÂNCIA DA MICROBIOTA NO TRANSPLANTE DE CÉULAS-TRONCO HEMATOPOIÉTICAS

### Microbiota intestinal

O TGI humano pode ser povoado por até 100 trilhões de bactérias (para comparação, o número de células no corpo humano é estimado em 10 trilhões), além de vírus e fungos presentes também em considerável número e diversidade, e que podem pertencer a aproximadamente mil espécies diferentes em um único indivíduo; já foram identificadas mais de 15 mil espécies diferentes em amostras derivadas de TGI humanos.^( 91 )^

O sistema imunológico do TGI é a primeira linha de defesa contra microrganismos e outras substâncias ingeridas, e evoluiu não apenas para a proteção contra potenciais patógenos, mas, também, para tolerar bactérias comensais que possuem um papel benéfico na homeostase, permitindo simbiose com a microbiota intestinal. O sistema imunológico gastrintestinal mantém a barreira mucosa por meio da secreção de peptídeos e anticorpos antimicrobianos, e a microbiota comensal participa da fisiologia intestinal do hospedeiro.^( 92 , 93 )^

A exposição intestinal a bactérias está relacionada ao recrutamento de linfócitos T regulatórios (Tregs).^( 94 , 95 )^As células Tregs são fundamentais para o desenvolvimento de uma resposta imunológica apropriada a antígenos dentro do TGI, mas também influenciam na imunidade sistêmica.^( 96 , 97 )^

As bactérias intestinais são responsáveis pela quebra de fibras da dieta, sendo também importantes para a produção de uma série de metabólitos com funções na fisiologia intestinal. Os mais bem conhecidos destes metabólitos são os ácidos graxos de cadeias curtas (SCFAs - *short chain fatty acids* ), como o butirato, o propionato e o acetato, que servem como fontes de energia para as células epiteliais intestinais e induzem respostas imunológicas regulatórias de proteção tanto localmente no TGI quanto sistemicamente.^( 98 , 99 )^

### Disbiose

A quimioterapia e os regimes de condicionamento alteram a composição da microbiota intestinal, provocando a redução de *Clostridium clusters* XIV e bifidobactérias, e o aumento de *Enterococcus* . Essa alteração da microbiota é chamada disbiose.^( 100 - 102 )^

Um estudo específico com pacientes submetidos ao TCTH encontrou níveis aumentados de proteobactérias, incluindo espécies de *Escherichia* e redução dos níveis de *Firmicutes* , incluindo espécies de *Blautia* após a quimioterapia.^( 102 )^

Entretanto, a relação causal entre quimioterapia e microbiota é difícil de ser estabelecida, pois muitos dos pacientes estudados receberam antibióticos profiláticos concomitantemente com a quimioterapia.

### Microbiota intestinal e doença do enxerto contra o hospedeiro

A microbiota intestinal normal apresenta grande diversidade e é dominada por bactérias anaeróbias.^( 103 )^Durante a internação, muitos pacientes submetidos ao TCTH perdem esta diversidade, e as mudanças que ocorrem são influenciadas tanto pelos tratamentos antimicrobianos quanto pelo desenvolvimento de DECH.^( 104 - 106 )^

O impacto da microbiota sobre a DECH foi inicialmente proposto nos anos 1970, após demonstração de que camundongos mantidos em condições livres de germes desenvolviam menos DECH de TGI.^( 107 , 108 )^Estudos clínicos subsequentes que evidenciaram resultados promissores na descontaminação intestinal de pacientes transplantados^( 109 , 110 )^não foram confirmados em pesquisas posteriores.^( 111 , 112 )^

Amplo estudo prospectivo focando a descontaminação de bactérias anaeróbicas evidenciou redução no desenvolvimento de DECH, indicando que a descontaminação seletiva poderia ter efeitos benéficos.^( 113 )^

A perda da diversidade intestinal observada em pacientes submetidos ao TCTH é geralmente associada à perda de espécies de *Clostridium* , que reconhecidamente produzem ácidos graxos de cadeia curta a partir das fibras da dieta.^( 114 )^

O butirato é a fonte de energia preferencial das células epiteliais intestinais, e um estudo sugere que quantidades reduzidas de butirato são encontradas nas células epiteliais intestinais de camundongos submetidos ao TCTH, e a adição destes ácidos graxos reduz as lesões intestinais e o desenvolvimento de DECH.^( 115 )^

Estes achados são reproduzidos pela administração de espécies variadas de bactérias produtoras de butirato pertencentes à classe *Clostridia* , e estudo clínico demonstrou que a abundância intestinal de bactérias do gênero *Blautia* , da classe *Clostridia* , correlaciona-se com risco reduzido de mortalidade devido à DECH.^( 116 )^

A administração de antibióticos para tratamento da neutropenia febril é, provavelmente, o principal fator que afeta as mudanças da microbiota observadas na evolução dos pacientes transplantados, e a escolha do regime de antibioticoterapia utilizado influencia na incidência de DECH. A administração de imipenem-cilastatina e piperacilina-tazobactam foi associada a maior mortalidade relacionada à DECH em 5 anos de seguimento, em um estudo retrospectivo.^( 117 )^Este mesmo estudo não demonstrou a associação entre metronidazol e redução de DECH reportado anteriormente,^( 113 )^o que pode ser devido a inúmeros fatores, incluindo o uso de combinações diferentes de antibióticos entre os estudos, bem como diferenças culturais e geográficas, que podem influenciar na flora intestinal.

A microbiota intestinal pode não apenas predispor a DECH, mas também atuar na recuperação e até na prevenção da doença. Os danos intestinais causados pelos regimes de condicionamento provocam aumento na permeabilidade intestinal que permite a translocação de bactérias por meio da barreira entérica. Como consequência, a estimulação imunológica por uma série de patógenos e moléculas associadas, como os lipopolissacarídeos e o peptidoglicano bacterianos, reforça a resposta inflamatória mediada por citocinas, propiciando o cenário ideal para a ativação alogênica dos linfócitos T.

O grau de perda da diversidade da microbiota intestinal é um fator de risco para a mortalidade relacionada ao transplante (MRT), incluindo a mortalidade por DECH, infecções e falências orgânicas após TCTH.^( 118 )^

## NUTRIÇÃO A MICROBIOTA

O uso de TNP reduz o montante de nutrientes que alcança o epitélio intestinal, e, dessa maneira, algumas das alterações na microbiota observada durante o TCTH podem decorrer da quantidade insuficiente de nutrientes no TGI para manter uma flora equilibrada.^( 116 )^

O estudo que evidenciou associação entre a redução de *Blautia* e a DECH também mostrou correlação entre este achado e prolongada TNP.^( 116 )^Estes achados sugerem que NE, ao contrário da TNP, pode exercer efeito benéfico na flora intestinal pós-TCTH e, talvez, acelerar a recuperação do paciente.

### Uso de probióticos e prebióticos

Uma atenção crescente tem sido dada ao potencial de probióticos e prebióticos na prevenção e no tratamento da disbiose intestinal. Os probióticos são suplementos nutricionais que contêm quantidade definida de microrganismos viáveis, cuja administração pode conferir benefícios ao paciente,^( 119 )^enquanto os prebióticos consistem em ingredientes alimentares não digeríveis (por exemplo, fibras não digeríveis), que favorecem o crescimento de bactérias benéficas.^( 119 )^

Até pouco tempo atrás, o uso de probióticos em indivíduos imunossuprimidos era totalmente proscrito, pois se acreditava que os mesmos, por serem bactérias vivas, poderiam causar doenças infecciosas graves. Porém, esse conceito vem sendo gradualmente modificado por vários estudos que demonstram, inicialmente, sua segurança nesse perfil de pacientes, além de potenciais efeitos de melhor prognóstico.

De maneira geral, vários estudos demonstram que o uso de probióticos em diversas condições clínicas - como nas doenças inflamatórias intestinais - é seguro pelo fato de serem indivíduos imunossuprimidos e também por estar relacionado à redução da resposta inflamatória sistêmica e local, por meio de adequada resposta imune; portanto, a indicação para o uso de probióticos em pacientes submetidos ao TCTH alogênico baseia-se nesta condição.^( 120 , 121 )^

Sabe-se que estes microrganismos podem inibir a atuação de patógenos externos; e melhoram a função de barreira intestinal, aumentando a produção de muco e de peptídeos com propriedades bactericidas, melhorando a estrutura das junções celulares entre os enterócitos e prevenindo apoptose celular precoce.^( 120 , 121 )^

Uma das cepas que mais tem sua segurança comprovada é o *Lactobacillus plantarum* (LPB). Além da segurança, também comprova-se *in vitro* que seu uso pré-TCTH diminui gravidade e mortalidade por DECH.^( 104 )^

Segundo Coehn et al., a análise retrospectiva de prontuários de 3.796 pacientes submetidos ao TCTH no período de 2002 a 2011, com o intuito de identificar infecção de corrente sanguínea por agentes probióticos ( *Lactobacillus, Bifidobacterium, Streptococcus thermophilus* e *Saccharomyces* ), evidenciou que apenas 0,5% (n=19) desenvolveu esta condição 1 ano após o transplante, sendo que, dos 19 doentes, 74% receberem TCTH alogênico, sendo 98% de infecção de corrente sanguínea por *Lactobaccilus* .^( 121 )^

Em 2004, Gerbitz et al., demonstraram em estudo experimental em ratos que o grupo tratado com *Lactobacillus rhamnosus* teve menor mortalidade que o grupo controle, principalmente no pós-TCTH recente (7 a 14 dias após infusão das células), além de apresentar manifestações mais brandas da DECH.^( 122 )^

Em 2015, Laval et al., publicaram outro estudo *in vitro* , considerando tanto a hipótese de que a permeabilidade das células intestinais está aumentada em várias doenças inflamatórias intestinais e inclusive na DECH, quanto a já comprovada teoria de que determinadas cepas probióticas podem aumentar a integridade intestinal. Nesse estudo, demonstram que o uso de *Lactobacillus rhamnosus* pode restaurar parcialmente a função de barreira dos enterócitos e também aumentar a produção de dipeptídeos protetores da mucosa intestinal.^( 123 )^

Em 2017, Gorshein et al., demonstraram, em estudo com 31 pacientes submetidos a TCTH alogênico que receberam *Lactbacillus rhamnosus* na dose diária de 10 bilhões de cepas, que seu uso é seguro e sem correlação com complicações infecciosas graves; porém, não foi evidenciada diferença estatística na morbimortalidade em ambos os grupos.^( 124 )^

Segundo Ladas et al., o uso de LPB é submetido à rigorosa análise microbiológica e, por isso, comprovadamente descontaminado, na dose de 1×108 colônias ofertada do dia -7 até o dia +14. Em estudo envolvendo 31 crianças e adolescentes (2 a 17 anos) submetidos ao TCTH alogênico com regime de condicionamento mieloablativo mostrou-se seguro, de forma que não foi observado episódio de bacteremia por LPB, bem como nenhuma outra complicação grave relacionada ao uso do LPB.^( 125 )^

Ainda segundo Ladas et al., 70% dos pacientes não desenvolveram DECH-a no d+100 e nenhum dos pacientes que vieram a óbito no d+100 desenvolveu DECH-a. Dos 30% que desenvolveram DECH-a, nenhum paciente apresentou gravidade máxima (grau 4).^( 125 )^

Embora o uso destes tratamentos pareça promissor, são necessários mais estudos clínicos para estabelecer a segurança e a eficácia dessas terapias. Um importante aspecto na eficácia do tratamento probiótico reside na capacidade de os microrganismos ingeridos sobreviverem ao ambiente ácido do estômago e do intestino delgado. Muitas cepas de lactobacilos, incluindo os mais comumente presentes em laticínios comuns, são sensíveis ao baixo pH gastrintestinal e não puderam ser reisolados em amostras fecais após administração em humanos,^( 126 )^dificultando a interpretação de sua eficácia.

O uso de probióticos e prebióticos no TCTH ainda não é recomendado de forma rotineira.

## TRANSPLANTE DE MICROBIOTA FECAL

O transplante de microbiota fecal (TMF) pode ser utilizado para restaurar uma flora intestinal danificada. Um estudo com pequena série de pacientes com DECH refratária ou dependente de corticosteroides apresentou resultados promissores;^( 127 )^no entanto, estudos maiores e mais bem controlados são necessários para determinar a eficácia do TMF no tratamento da DECH.

O TMF para o tratamento de infecções por *Clostridium difficile* resistentes já é uma técnica bem descrita em diversas populações.^( 128 )^Seu uso ainda é modesto no contexto de pacientes pós-transplante, e uma das experiências pioneiras foi feita no Brasil, sem que houvesse maiores complicações.^( 129 )^Desde então, outros casos já foram relatados com sucesso, utilizando doadores familiares ou não, e usando alguns métodos, como enteroscopia retrógrada ou a ingestão de cápsulas que se abrem somente no jejuno, liberando a nova microbiota.^( 130 )^

No momento da publicação deste consenso, o TMF para fins de imunomodulação e tratamento da DECH é algo promissor, mas que só deve ser feito dentro de estudos clínicos bem delineados. É necessário entender quais componentes da microbiota são desejáveis, assim como saber qual seria o melhor momento de realizar esse tipo de intervenção. Já seu uso no tratamento de infecções por *Clostridium difficile* , embora careça de ensaios randomizados e grandes séries de casos nesse grupo de pacientes, pode ser considerado em situações especiais, já que, até o momento, não foram relatadas complicações.

## UM ORGANOGRAMA PRÁTICO

Abaixo as figuras 1 e 2 resumem de modo prático as condutas nutricionais na DECH.

**Figura 1:** Planejamento nutricional ao transplante de celulas-tronco hematopoiéticas TCTH: transplante de celulas-tronco hematopoiéticas; TNO: terapia de nutrição oral; TNE: terapia de nutrição enteral; TNP: terapia de nutrição parenteral.

**Figura 2:** Planejamento nutricional da doença do enxerto contra o hospedeiro do trato gastrintestinal DECH: doença do enxerto contra o hospedeiro; TGI: trato gastrintestinal.
